# Binding of α-synuclein oligomers to Cx32 facilitates protein uptake and transfer in neurons and oligodendrocytes

**DOI:** 10.1007/s00401-019-02007-x

**Published:** 2019-04-11

**Authors:** Juan F. Reyes, Christopher Sackmann, Alana Hoffmann, Per Svenningsson, Jürgen Winkler, Martin Ingelsson, Martin Hallbeck

**Affiliations:** 1 Department of Clinical Pathology and Department of Clinical and Experimental Medicine, Linköping University, Linköping, Sweden; 2 Department of Molecular Neurology, University Hospital Erlangen, Erlangen, Germany; 3 Department of Clinical Neuroscience, Karolinska Institute, Stockholm, Sweden; 4 Section of Geriatrics, Department of Public Health and Caring Sciences, Uppsala University, Uppsala, Sweden

**Keywords:** Parkinson’s disease (PD), Multiple system atrophy (MSA), Alzheimer’s disease (AD), Cell-to-cell transfer, Prion-like transfer, Gap junction proteins, Cx32, GJB1, alpha-Synuclein (α-syn)

## Abstract

The intercellular transfer of alpha-synuclein (α-syn) has been implicated in the progression of Parkinson’s disease (PD) and multiple system atrophy (MSA). The cellular mechanisms underlying this process are now beginning to be elucidated. In this study, we demonstrate that the gap junction protein connexin-32 (Cx32) is centrally involved in the preferential uptake of α-syn oligomeric assemblies (oα-syn) in neurons and oligodendrocytes. In vitro, we demonstrate a clear correlation between Cx32 expression and oα-syn uptake. Pharmacological and genetic strategies targeting Cx32 successfully blocked oα-syn uptake. In cellular and transgenic mice modeling PD and MSA, we observed significant upregulation of Cx32 which correlates with α-syn accumulation. Notably, we could also demonstrate a direct interaction between α-syn and Cx32 in two out of four human PD cases that was absent in all four age-matched controls. These data are suggestive of a link between Cx32 and PD pathophysiology. Collectively, our results provide compelling evidence for Cx32 as a novel target for therapeutic intervention in PD and related α-synucleinopathies.

**Electronic supplementary material:**

The online version of this article (10.1007/s00401-019-02007-x) contains supplementary material, which is available to authorized users.

## Introduction

Parkinson’s disease (PD) is clinically characterized by the progressive decline of motor and non-motor functions [47]. The progressive accumulation of hallmark protein deposits comprised primarily of the alpha-synuclein protein (α-syn) is a signature lesion of PD and other neurodegenerative disorders which collectively are known as synucleinopathies [16]. These pathological conditions include dementia with Lewy bodies (DLB) and multiple system atrophy (MSA), which, in addition to PD, are the most common synucleinopathies [57, 58]. Rare mutations (A30P, E46K, and A53T) or multiplications of the α-syn gene (*SNCA*) have been linked to autosomal dominant forms of PD that promote early disease onset [15, 24, 36]. In contrast to PD and DLB, where α-syn accumulation occurs predominantly in neurons, the accumulation of α-syn in cells of the oligodendrocyte lineage is the characteristic pathological feature of MSA [67]. While α-syn aggregation in neurons is limited in MSA, a widespread neuronal degeneration is still observed and may partly account for the progressive clinical symptoms that in some instances resemble those of PD [67]. To date, accumulating evidence has shown that pathological α-syn has the capacity to self-seed, aggregate, and propagate between cells in vitro and in animal models of PD and MSA [3, 10, 25, 27, 70]. While the molecular mechanisms underlying α-syn cell-to-cell transfer are only beginning to be understood, a direct neuroanatomical connection has been suggested to play a role [28, 35, 41, 42]. Moreover, the recent identification of two novel receptors specific for α-syn fibrils (LAG3 and FcγRIIB) [7, 29] has shed light on the potential existence of membrane receptors for α-syn oligomers, protein assemblies associated with neurotoxic properties and cellular propagation [20].

Direct cellular connectivity and communication are provided in part by dynamic multifunctional membrane structures termed gap junction channels (GJC). These structures are composed of two opposing hemichannels, each formed by the self-assembly of six transmembrane proteins termed connexins (Cxs) [4]. In the human brain, 21 Cx genes have been shown to be translated into proteins that differentially interact to generate multiple GJC types (homomeric or heteromeric). As such, each GJC type acquires different functional properties that depend on the Cx proteins involved [5, 71]. Under normal physiological conditions, GJC and hemichannels play independent, and critical roles to maintain and promote intercellular homeostasis, allowing the direct transfer of ions and small molecules less than 1 kDa in size (e.g., sugars, amino acids, and nucleotides) between connecting cells [75]. However, dysregulation of Cx expression coupled with Cx-mediated uptake and transfer of nonconventional proteins have been reported in multiple pathological conditions, including viral infections and neurodegenerative disorders [49]. For example, Cx dysregulation mediated by oligomeric amyloid-beta peptides (oAβ) associated with Alzheimer’s disease (AD) [33] as well as the viral transfer of thymidine kinase [61] and dickkopf-1 proteins from herpes simplex and human immunodeficiency viruses [32], respectively, has been described.

In the current study, we report the identification of Cx32 centrally involved in the selective uptake and propagation of α-syn oligomers (oα-syn) in both neurons and oligodendrocytes, the primary cell types affected by α-syn aggregation in PD and MSA, respectively. Correspondingly, modulation of Cx32 protein turnover using pharmacological and genetic strategies demonstrates a clear association between Cx32 expression and oα-syn uptake. Moreover, we found that recipient cells overexpressing Cx32 more readily take up oα-syn from donor cells expressing α-syn-GFP relative to control cells expressing untagged mCherry proteins in a co-culture system [43]. By blocking Cx32 activity using either Cx32-specific peptide mimetics or selective gap junction inhibitors, we decreased oα-syn uptake in a concentration-dependent manner. Furthermore, we found that exposure to oα-syn assemblies or expression of the α-syn gene (*SNCA*) promotes Cx32 upregulation. Consistent with our in vitro findings, we identified a significant upregulation of Cx32 in transgenic (Tg) animal models of PD (L61, A30P) and MSA (MBP29) overexpressing human α-syn in neurons or oligodendrocytes, respectively. Finally, we could demonstrate a direct interaction between α-syn and Cx32 in two out of four human PD cases that was absent in all four age-matched controls, suggesting a potential link between Cx32 and PD pathophysiology. Collectively, our results provide strong evidence for Cx32 centrally involved in the preferential uptake and propagation of oα-syn assemblies, pinpointing Cx32 as a novel therapeutic target to impede the uptake and spread of α-syn pathology in PD and related α-synucleinopathies.

## Materials and methods

### Generation of ATTO-labeled α-syn

Recombinant α-syn protein was purchased in a lyophilized form from Alexotech in a lyophilized form. To covalently label α-syn with ATTO-550 or ATTO-488 fluorescent tags, we used a protein:dye ratio of 1:2 as previously reported [45]. The protein solution was then mixed with reactive ATTO-550 dye freshly dissolved in DMSO (Sigma-Aldrich). Unreactive dye was removed by gel filtration chromatography using GE Sephadex G-25 (GE Healthcare), and labeled fractions were analyzed by Western blot analysis and size exclusion chromatography (SEC). Positive fractions were pooled, lyophilized, and then stored at − 20 °C until further use.

### Generation of α-syn and amyloid-beta (Aβ) assemblies

To generate different α-syn assemblies, we solubilized ATTO-labeled proteins in PBS to generate soluble monomers at a concentration of 4.0 mg/mL. We then separated α-syn monomers into three different fractions, each containing 350 μL of protein per tube, thereby ensuring the same concentration for all α-syn assemblies. The monomeric fraction was immediately stored at − 80 °C until further use. To generate oligomers and fibrils, we incubated monomers at 37 °C for 5 days in an Eppendorf SS mini-shaker (Eppendorf) with constant shaking at either 350 or 1000 RPM, respectively. Assembled proteins were then aliquoted and stored at − 80 °C until further use. To generate oAβ assemblies, we prepared recombinant Aβ peptide (1–42) as previously reported [55]. Briefly, recombinant Aβ1–42 peptides (Innovagen) were dissolved in 1,1,1,3,3,3-hexafluoro-2-propanol (HFIP) and vacuum-dried overnight. The next day, Aβ1–42 was diluted to a final concentration of 100 uM in HEPES (20 mM pH 7.4), vortexed, sonicated for 10 min, and incubated at 4 °C overnight to generate oligomeric assemblies.

### Transmission electron microscopy (TEM) characterization of α-syn assemblies

α-syn monomers, oligomers, and fibrillar assemblies were prepared for TEM analysis as previously reported [45]. Briefly, each sample was placed on carbon-coated 200-mesh grids, negatively stained with 1% uranyl acetate, and analyzed with a Jeol 1400 transmission electron microscope (Jeol). Images were taken using a Gatan Orius CCD camera (Gatan).

### Size exclusion chromatography (SEC)

SEC was performed to confirm protein assembly as previously reported [11]. Briefly, protein samples were loaded on a Superdex 75 10/300 GL column (GE Healthcare), and eluted protein fractions were measured using absorbance of 280 nm. For confirmation of ATTO-550 labeling, protein fractions were also analyzed at an absorbance of 550 nm.

### Human-induced oligodendrocyte progenitor cells (OPC) differentiation

To differentiate human induced pluripotent stem cells derived OPCs (Tempo Biosciences) into mature, myelin-positive oligodendrocytes (MBP) we plated OPCs on matrigel (Corning) and incubated them with DMEM/F12 containing HEPES, glutamine (2 mM), nonessential amino acids (1X) (Life Tech), 1X StemPro Neural Supplement (Invitrogen), PDGF-AA (10 ng/mL, Cat: 100-13A, Peprotech), NT3, (10 ng/mL, Cat: 450-03, Peprotech), biotin (100 ng/mL, Sigma-Aldrich), and cAMP (5 µM, Sigma-Aldrich). Cell differentiation was induced for 30 days following incubation with 50:50 DMEM/F12:Neurobasal medium (Life Tech) containing nonessential amino acids 1X (Life Tech), 1X B27 (Life Tech), 2 l-glutamine (Life Tech), biotin (100 ng/mL, Sigma-Aldrich), brain-derived neurotrophic factor (BDNF, 10 ng/mL; Cat: 450-02, Peprotech), ascorbic acid (20 μg/mL, Sigma-Aldrich), cAMP (1 μM, Sigma-Aldrich), and T3 (200 ng/mL, Sigma-Aldrich).

### Differentiation of human SH-SY5Y cells and rat OLN-93 oligodendrocytes

SH-SY5Y cells (ECACC: Sigma-Aldrich) and OLN-93 oligodendrocytes (a gift from Professor Christiane Richter-Landsberg at the University of Oldenburg, Oldenburg, Germany) were cultured in modified Eagle’s medium (MEM) containing glutamine and supplemented with 10% fetal bovine serum (FBS) (Life Technologies), 100 U/mL penicillin, and 100 µg/mL streptomycin (Lonza) in a humidifier incubator at 37 °C with 5% CO_2_. SH-SY5Y and OLN-93 differentiation was performed as previously reported [11, 45]. Briefly, SH-SY5Y cells were cultured in 12-well plates for 7 days using 10 µM retinoic acid (RA) to induce a pre-differentiated state. Following RA differentiation, the medium was changed to MEM without FBS supplemented with BDNF (50 ng/mL, PeproTech), neuregulin β1 (10 ng/mL, R&D Systems), nerve growth factor (10 ng/mL, R&D Systems), and vitamin D_3_ (24 nM, Sigma-Aldrich). OLN-93 oligodendrocyte differentiation was performed by incubating cells in MEM without serum containing glutamine and with 1% penicillin/streptomycin without FBS (Life Technologies).

### Generation of SH-SY5Y, OLN-93, and OPC cell lines

Stable human SH-SY5Y, rat OLN-93 cell lines, and human OPC expressing proteins of interest were generated by transfection with plasmid vectors expressing either human Cx32-mCherry (a gift from Michael Davidson, Addgene plasmid # 55022), Cx43–IRES–GFP (a gift from Trond Aasen, Addgene plasmid # 65433), Cx43-HA (a gift from Robin Shaw, Addgene plasmid # 49851), mCherry (a gift from Michael Davidson, Addgene plasmid # 54517), and α-syn-GFP using an empty GFP plasmid vector (a gift from Didier Trono, Addgene plasmid # 12258) according to the manufacturer’s instructions (Amaxa Nucleofector, Lonza). To generate Cx32-KO cell lines, we nucleofected cells (Amaxa Nucleofector, Lonza) with commercially available gRNA plasmid sequences (Santa Cruz Biotechnology, Cat# 2234) or self-generated sequences containing a fluorescent reporter. When applicable, transfected cells were selected with appropriate antibiotics (G418, Sigma-Aldrich) followed by single cell sorting using FACS into 96-well plates to generate clonal cell lines.

### HEK-293 transfection and α-syn treatment

HEK-293 cells were seeded in a 6-well plate at 90% confluency prior to transfection. The next day, we transfected the cells with either Cx32 or Cx43 plasmids (as above) using Lipofectamine^®^ LTX reagents according to the manufacturer’s instructions (Life Technologies) with increasing concentrations of plasmid DNA constructs (15 and 50 µg, respectively). Briefly, 1 h prior to transfection, proliferation medium (MEM with serum) was removed and replaced with MEM containing glutamine and 1% penicillin/streptomycin without FBS. Subsequently, the growth medium was removed and replaced with MEM containing plasmid DNA and transfection reagents. The next day, cells were trypsinized and transferred to a 12-well plate for α-syn uptake analysis. Cells were then allowed to attach prior to treatment with different α-syn assemblies (1 µM) in serum free MEM medium (with glutamine and 1% penicillin/streptomycin) for 24 h. Cells were then washed three times in PBS, trypsinized and pelleted by centrifugation at 13,000 RPM for 5 min on a benchtop centrifuge (Eppendorf) then prepared for Western blot analysis.

### Primary neuron isolation

Primary cortical neurons were obtained from mouse cortices on embryonic day 16 [8]. In brief, cortices were dissected, and neurons were seeded onto 12-well culture dishes at 3 × 10^5^ cells/well in Neurobasal media (Life Technologies) supplemented with penicillin (5 U/mL), streptomycin (5 g/mL), B27 supplement, and glutamine.

### Pharmacological gap junction inhibition and peptide mimetic treatments

To pharmacologically inhibit gap junction proteins, we treated differentiated SH-SY5Y cells, primary neurons (DIV 10) and differentiated OLN-93 oligodendrocytes for 24 h in the presence of increasing concentrations of CBX (50, 100 µM), MQ (25, 50 µM), 2-APB (100 or 200 µM, Sigma-Aldrich) or the synthetic peptide mimetics Gap3211, Gap2409, and Gap2605 (100, 200 µM, Alpha Diagnostic International) in the presence of 1 µM ATTO-550 labeled α-syn assemblies (oligomers of fibrils) for 24 h in MEM with glutamine and 1% penicillin/streptomycin without FBS (Life Technologies). Following pharmacological or peptide mimetic treatment, cells were washed three times with PBS followed by fixation with 4% paraformaldehyde (PFA) for 20 min. Cells were then washed and prepared for immunocytochemistry.

### Inhibition of the p38 MAPK pathway

To inhibit or activate the p38 MAPK pathway, we treated differentiated human SH-SY5Y cells expressing α-syn-GFP with increasing concentrations of the MAPK inhibitor SB203580 (10 and 25 µM) or anisomycin (1, 3, and 5 µM, cell-signaling technology), respectively, in the presence of MEM (without FBS) containing α-syn oligomers (1 µM) for 24 h at 37 °C. Following treatment, cells were washed three times with PBS and subjected to Western blot or RT-PCR analysis.

### Cellular energy assessment analysis

To assess whether Cx32-mediated uptake is an energy-dependent mechanism, we treated differentiated Cx32-mCherry- or mCherry-expressing cells with 1 µM oα-syn. Following protein treatment, we incubated cells at either 37 °C or 4 °C for 6 h. Cells were then washed with PBS and subjected to Western blot analysis.

### Immunocytochemistry and immunohistochemistry

Differentiated SH-SY5Y cells, primary neurons, and differentiated OLN-93 cells were processed for immunocytochemistry as previously reported [45]. Briefly, cells fixed with 4% PFA were washed three times with PBS and permeabilized by incubation in 0.1% Triton X-100 in PBS for 30 min on ice. Next, cells were washed with PBS and incubated in 5% BSA for 1 h at room temperature, followed by incubation with primary antibodies overnight at 4 °C (Suppl. Table S1, Online Resource 1). The next day, we washed the cells with PBS and incubated them with respective fluorescently labeled secondary antibodies for 1 h (goat anti-mouse or goat anti-rabbit IgG Alexa Fluor 488, 564 or 633, Life Technologies), followed by Hoechst staining (Sigma-Aldrich). For immunohistochemistry, paraffin-embedded tissue sections were rehydrated in xylene (Sigma-Aldrich) followed by incubation in decreasing concentrations of ethanol (100, 95, 70, and 50%) then rinsed in water for 10 min. Epitope retrieval was then performed using acidic conditions according to the manufacturer’s instructions (Dako). Tissue sections were rinsed three times (5 min) with 0.04% Triton-X in PBS and processed for immunohistochemistry as described above.

### Confocal image analysis

To visualize the fluorescence immunostaining, we used a Zeiss LSM 510 confocal microscope equipped with Ar and HeNe lasers to acquire Z-stacks of consecutive confocal images. For fluorescence measurements, all imaging parameters (e.g., laser power, exposure, and pinhole size) were held constant after microscope settings were established using appropriate controls. The results are provided as the relative fluorescent intensity of drug-treated samples compared to untreated controls.

### Brain homogenate sample preparation

Mouse brain hemispheres from Tg animals overexpressing wild-type α-syn in neurons (Line 61) and wild-type littermate controls were purchased from QPS-neuro, Austria [46]. Brain samples from mice harboring mutant human α-syn proteins (A30P, Suppl. Table S2, Online Resource 2) were generated and obtained from Professor Philipp Kahle [23]. Frozen cortical regions were separated from hemispheres and then homogenized in RLT lysis buffer (Qiagen) on ice using a hand-held homogenizer (Polytron, PT 2100, Kinematica AG). We then briefly sonicated (SoniPrep 150) samples to eliminate non-homogenized clumps and centrifuged them at 1000 RPM for 5 min. The supernatant was collected and quantified using Nanodrop (Saveen and Werner), and prepared for Western blot analysis and qRT-PCR. For human brain samples (Suppl. Table S3, Online Resource 3), frozen tissue sections from the putamen or SNpc isolated from controls or PD cases were obtained from Karolinska Institute (KI) and processed as described above. The MSA mouse model (MBP29-hα-syn mice, hereafter: MBP29 mice) was obtained from Professor Eliezer Masliah [56]. All animals were treated according to the European Union (2010/63/EU) and NIH (National Institute of Health) guidelines for the humane treatment of animals. Cohorts were maintained under standard animal housing conditions with a 12 h dark–light cycle and free access to food and water. Mice were euthanized under anesthesia and transcardially perfused with 0.9% sodium chloride solution. Cortices and corpora callosa were then microdissected and homogenized using QIAzol Lysis Reagent (Qiagen) for tissue homogenization containing proteinase and phosphatase inhibitors. Sonication was performed on ice for 20 s and the supernatant was collected after centrifugation. The addition of chloroform was followed by centrifugation-induced phase separation. Samples were further diluted in radioimmunoprecipitation assay (RIPA) buffer, and the protein concentration was measured using the Pierce™ BCA protein assay kit (Thermo Scientific). The human MSA samples and age as well as gender-matched controls (obtained from the Netherlands Brain Bank, Netherlands Institute for Neuroscience, Amsterdam)—were also prepared as described above. RNA was purified using an RNeasy mini kit (Qiagen) according to the manufacturer’s instructions, and the concentration was determined using Nanodrop (PeqLab). The GoScript™ Reverse Transcription System (Promega) was used for cDNA generation, and gene transcription was quantified in a Light Cycler 480 (Roche) using SSo Fast EvaGreen Supermix (Bio-Rad).

### Western blot analysis

Following exogenous protein treatment, cells were washed three times in PBS for 5 min and subsequently incubated with trypsin for 5–10 min until complete cellular detachment. Cells were then pelleted by centrifugation at 13,000 RPM (Eppendorf) and lysed in SDS-lysis buffer followed by a brief sonication to completely lyse cell pellets and determine protein concentration using DC Assay (Bio-Rad). All samples were then resuspended in a final concentration of 1X Laemmli’s sample buffer, boiled for 5 min, separated on 10% SDS-PAGE (CBS Scientific), and transferred to nitrocellulose membranes using iBLOT kits (Life Technologies). Non-specific protein binding was blocked by incubating membranes with 2% nonfat dry milk, followed by incubation with primary antibodies (Suppl. Table S1, Online Resource 1) at 4 °C overnight. After rinsing the membranes using Tris-buffered saline (Life Technologies), we incubated them with peroxidase-conjugated goat anti-mouse or anti-rabbit IgG H + L secondary antibodies (Dako) for 1 h at room temperature, followed by ECL substrate (Bio-Rad) to visualize the signal on a ChemiDoc XRS+ (Bio-Rad). Densitometric analysis was performed using ImageJ (Fiji), and values with arbitrary units were normalized to the signals obtained from total protein measured with either GAPDH or β-actin (Suppl. Table S1, Online Resource 1). For MSA samples, proteins were resuspended in a final concentration of 1XNuPAGE™ LDS Sample buffer (Thermo Scientific) and 1X NuPAGE™ Sample Reducing Agent, heated for 10 min at 70 °C and separated on NuPAGE™ 4–12% Bis–Tris Protein Gels (Thermo Scientific). Afterwards, proteins were transferred to an EMD Millipore Immobilon™-FL PVDF Transfer Membrane (Millipore). Non-specific protein binding was blocked by incubating membranes with 1% BSA in Tris-buffered saline supplemented with 1% Tween-20 (TBS-T). The primary antibody was diluted in blocking buffer and incubated at 4 °C overnight. The next day, membranes were washed three times in TBS-T and incubated with the secondary antibodies donkey anti-mouse or donkey anti-rabbit diluted in blocking buffer for 1 h at room temperature. Membranes were washed three times with TBS-T and completely dried. Fluorescent signals were detected using FusionFX (Peqlab). Densitometric analysis of specific bands was performed using Bio-1D software (Vilber Lourmat), and values with arbitrary units were normalized to the signal obtained from total protein measured with β-actin.

### Immunoprecipitation of α-syn

α-Syn proteins were precipitated according to a previous report [60]. Briefly, cell lysates or frozen brain tissue blocks from controls and PD cases were homogenized in lysis buffer containing 20 mM Tris–HCl (pH 7.9), 137 mM NaCl, 5 mM N Na_2_EDTA, 1 mM EGTA, 10% glycerol, and 1% Triton X-100 with protease and phosphatase inhibitors (Roche). For cell lysates, 500 µg and 2 mg of human brain extract were incubated with 1 µg and 4 µg of antibody, respectively, overnight at 4 °C. A 1:1 suspension of Protein A/G agarose beads was added (Thermo Scientific), and the mixture was incubated at 4 °C for 4 h. The beads were then pelleted and washed thoroughly with cell lysis buffer according to the manufacturer’s instructions. Bound proteins were separated by SDS-PAGE followed by Western blot analysis.

### Co-culture conditions and FACS

We co-cultured “donor” and “recipient” cells (1:1 ratio) under proliferating (T-25 flasks) or differentiating conditions (12-well plates) at 37 °C with 5% CO_2_ as previously reported [2, 43]. After trypsinization, the cells were washed three times in PBS and passed through a 40-μm filter prior to flow cytometry analysis. To block cellular connectivity, we co-cultured donor and recipient cells as above (1:1) in 6-well plates separated by 40-μm porous transwell inserts (Falcon) that allow media sharing, but prevent cellular connectivity. After 5 days of incubation, we prepared recipient cells (Cx32-mCherry or mCherry) for flow cytometry and assessed cell viability using SytoxRed according to the manufacturer’s instructions (Life Technologies) as previously reported [43]. All samples were analyzed on a BD FACSAria III with DIVA software (BD Bioscience) through a 100-μm nozzle at a flow rate of 300–500 cells/min until 5 × 10^4^ cells per sample were analyzed.

### Confocal image analysis of FACS-sorted cells

Co-cultured cells were loaded on a FACS Aria III with DIVA software (BD Biosciences) and double-positive cells were plated onto poly-l-lysine (Thermo Scientific) coated glass microscope chamber slides (Lab-tek), and allowed to attach overnight. We then fixed the cells in 4% PFA for 15 min at room temperature, washed them three times with PBS, permeabilized with 0.01% Triton X-100, and stained for Hoechst (Sigma-Aldrich). Cells were analyzed with a Zeiss LSM 510 confocal microscope equipped with Ar and HeNe lasers. Protein colocalization was analyzed by calculating the Pearson correlation coefficient from confocal images using Huygens Pro software (Hilversum, Netherlands).

### Semi-quantitative real-time PCR (qRT-PCR)

RNA was extracted from cell and tissue samples using RNeasy Mini Kits according to the manufacturer’s instructions (Qiagen). cDNA was generated from RNA using a High-Capacity RNA to cDNA Kit (Applied Biosystems). Each RT-PCR reaction utilized 25 ng cDNA and was analyzed in technical duplicates or triplicates. Reactions were analyzed on a 7500 Fast Real-Time PCR System (Applied Biosystems). All primer/probes used in this study utilized the FAM–MGB TaqMan system and were purchased from Applied Biosystems or were generated using the primer design tool Primer-BLAST (http://www.ncbi.nlm.nih.gov/tools/primer-blast) (Suppl. Table S1, Online Resource 1).

### Statistical analyses

Every reported *n* is the number of biologically independent replicates. Except when noted otherwise, statistical analyses were performed using a one or two-way ANOVA with Tukey’s test when comparing multiple samples and genotypes and a two-tailed, unpaired Student’s *t* test when comparing two genotypes. Data distribution was assumed to be normal, but this was not formally tested. For Western blot analyses, band intensities were quantified using ImageJ software (Fiji) or Bio-1D (Vilber Lourmat) software, and values with arbitrary units were normalized to the signal obtained from the protein loading control. In each data group, the results are expressed as the mean ± SEM. For semi-quantitative RT-PCR, statistics were calculated using 2^−ddCt^ values, using One-way ANOVA with Tukey analysis for multiple comparisons. All data analyses were performed with GraphPad Prism 7.0 (La Jolla, CA). Findings were regarded as significant when * *p *< 0.05, **p < 0.01, ***p < 0.001, ****p < 0.0001.

## Results

### Cx32 facilitates the uptake of oα-syn assemblies in vitro

**Fig. 1:**
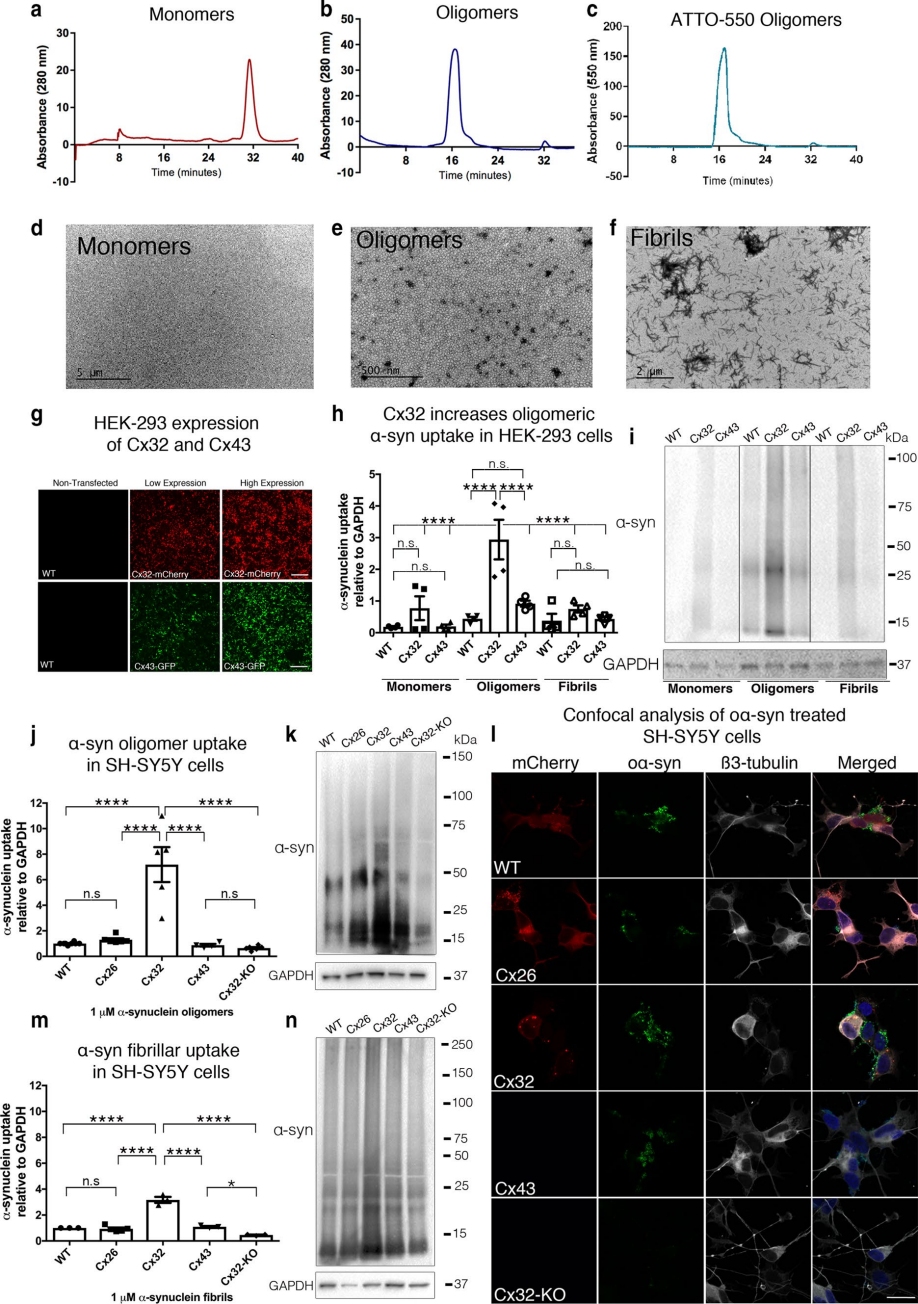
Cx32 facilitates the uptake of oα-syn preferentially to monomers or fibrillar assemblies. **a** SEC analysis of α-syn monomers or **b** oligomeric α-syn (oα-syn) assemblies at 280 nm absorbance. **c** SEC confirmation of labeled ATTO-550 oα-syn assemblies at 550 nm absorbance. **d** TEM characterization of α-syn monomers, **e** oligomers, or **f** fibrillar assemblies; scale bars represent 5 µm, 500 nm, and 2 µm, respectively. **g** Confocal image analysis of non-transfected wild-type (WT) or transfected HEK-293 cells with low (15 µg) or high (50 µg) expression of Cx32-mCherry or Cx43-GFP plasmid constructs. Scale bars represent 200 µm. **h** Densitometric analysis of **i** Western blot of monomeric, oligomeric or fibrillar α-syn uptake in WT HEK-293 cells or HEK-293 cells expressing Cx32 or Cx43 (*n* = 4, two-way ANOVA followed by Tukey’s post hoc test for multiple comparisons, n.s. = no significance, *F*_(8, 24)_ = 13.1, ****p < 0.0001). **j** Densitometric analysis of **k** representative Western blots of oα-syn uptake in differentiated SH-SY5Y WT cells or SH-SY5Y cells expressing Cx26, Cx32, Cx43 or Cx32-KO, (*n* = 5, one-way ANOVA followed by Tukey’s post hoc test for multiple comparisons, n.s; no significance, *F*_(4, 24)_ = 25.28, ****p < 0.0001). **l** Immunocytochemistry of differentiated SH-SY5Y cells, WT and those expressing Cx26, Cx32, Cx43 or Cx32-KO treated with oα-syn assemblies. Scale bars represent 20 µm. **m** Densitometric analysis of **n** representative Western blot of fibrillar α-syn uptake in differentiated SH-SY5Y cells, WT and those expressing Cx26, Cx32, Cx43, or Cx32-KO (*n* = 3, one-way ANOVA followed by Tukey’s post hoc test for multiple comparisons, n.s; no significance, *F*_(4, 12)_ = 76.16, *p < 0.05, ****p < 0.0001)

To determine whether connexins play a role in the uptake of different α-syn assemblies, we first generated recombinant α-syn monomers, oligomers (oα-syn), and fibrillar assemblies fluorescently tagged with ATTO-550 [45]. We then characterized soluble protein assemblies by size exclusion chromatography (SEC) as previously reported (Fig. 1a, b) [45]. As expected, we observed prolonged retention values for oα-syn assemblies compared to α-syn monomers. The absence of free dye within our preparations was corroborated by measuring the absorbance of ATTO-550 (550 nm) using SEC (Fig. 1c). We next characterized all protein assemblies by transmission electron microscopy (TEM) and concomitant to the prolonged SEC retention values observed, the characteristic donut-like and filament ultrastructures typical of oα-syn and fibrillar assemblies, respectively, were identified (Fig. 1d–f and Suppl. Figure S1a, b, Online Resource 4) [45]. We next analyzed the fibril length by TEM and observed an average length of 133.74 nm ± 3.53 nm, a result consistent with the length of non-sonicated α-syn fibrils (Suppl. Figure S1c, Online Resource 4) [44]. Following protein characterization, we transiently transfected human embryonic kidney cells (HEK-293) with increasing concentrations of the neuronal, non-canonically expressed Cx32 or the non-neuronally associated Cx43 (fused to mCherry and GFP, respectively), to exclude a general Cx effect. As expected, increased concentration of plasmid DNA transfection (15 µg; low and 50 µg; high) enhanced Cx expression in HEK-293 cells as shown by the increased intensity of the fluorescent tags observed under confocal microscopy and densitometry analysis (Fig. 1g and Suppl. Figure S1d, Online Resource 4). Consistent with the previous observations [40], the addition of fluorescent tags had no effect on Cx activity, gap junction formation or membrane compartmentalization, as shown by the subcellular localization of Cx32-mCherry or Cx43-GFP at the cellular membrane (Suppl. Figure S1e, f, Online Resource 4). Interestingly, treatment of Cx-expressing cells with α-syn monomers, oligomers, or fibrillar assemblies demonstrated a selective increase in the uptake of oα-syn that was Cx32-dependent (Fig. 1h, i). In contrast, HEK-293 cells expressing Cx43 showed no differences in protein uptake regardless of the assembly state of the α-syn molecule (Fig. 1h, i). Moreover, the increased levels of Cx32 in high-expression cells further amplified oα-syn uptake, suggesting a correlation between Cx32 expression and oα-syn uptake (Suppl. Figure S1d, g, Online Resource 4).

To verify that Cx32-mediated oα-syn uptake was not due to differential expression in HEK-293 cells but a Cx32-dependent mechanism, we generated multiple clonal SH-SY5Y cell lines expressing Cx32 (Cx32-mCherry) or its heteromeric gap junction binding partner Cx26 (Cx26-mCherry) [59]. We also generated clonal cell lines expressing Cx43 (Cx43-HA) as well as untagged mCherry proteins alone to use as controls. The contribution of Cx32 to oα-syn uptake was further tested by deletion of the Cx32 gene *(GJB1)* using CRISPR/Cas9 (Cx32-KO). Following SH-SY5Y differentiation to generate a neuron-like phenotype with morphological and biochemical characteristics of mature neurons [2, 73], we observed Cx32 expression localized to the cellular membrane. We next incubated SH-SY5Y cells with α-syn monomers, oligomers or fibrillar assemblies for 24 h. Consistent with the HEK-293 results (Fig. 1h, i), expression of Cx32 in differentiated neuronal SH-SY5Y cells selectively increased the uptake of oα-syn as analyzed by Western blot and immunocytochemistry (Fig. 1j–l) when compared to α-syn monomers (Suppl. Figure S1 h, Online Resource 4) and fibrillar assemblies (Fig. 1m, n). Consistent with the involvement of Cx32 in oα-syn uptake, CRISPR/Cas9-based deletion of the Cx32-mCherry construct significantly blocked oα-syn uptake, validating the involvement of Cx32 in the uptake of oα-syn assemblies (Suppl. Figure S1i, Online Resource 4). As expected, overexpression of Cx26 or Cx43 in differentiated SH-SY5Y cells showed no differences in the uptake of any α-syn assemblies, corroborating a Cx32-dependent mechanism in the selective uptake of oα-syn assemblies (Fig. 1j–n).

### The mitogen-activated protein kinase (MAPK) pathway modulates oα-syn uptake via Cx32

**Fig. 2:**
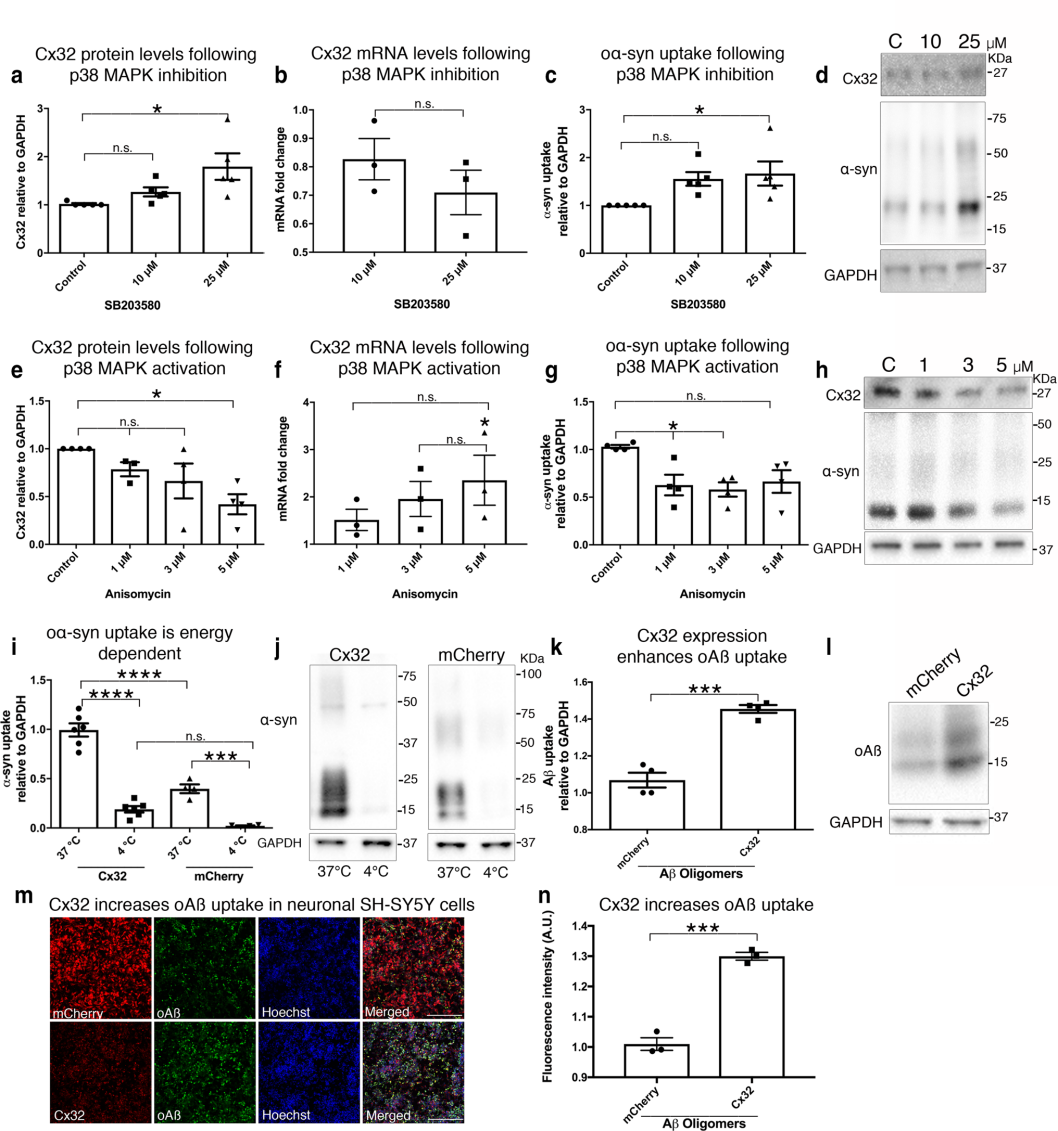
Cx32 expression correlates with oα-syn uptake. **a** Densitometric analysis of Cx32 protein levels in differentiated SH-SY5Y cells exposed to SB203580 for 24 h (10 and 25 µM), (*n* = 5, one-way ANOVA followed by Tukey’s post hoc test for multiple comparisons, n.s; no significance, *F*_(2, 12)_ = 5.547, *p < 0.05). **b** qRT-PCR analysis of Cx32 mRNA in differentiated SH-SY5Y cells with increasing SB203580 concentrations and untreated cells as controls (*n* = 3, one-way ANOVA followed by Tukey’s post hoc test for multiple comparisons, n.s. = no significance *F*(_3, 8_) = 1.415). **c** Densitometric analysis of oα-syn uptake in differentiated cells exposed to SB203580 for 24 h (*n* = 5, one-way ANOVA followed by Tukey’s post hoc test for multiple comparisons, n.s; no significance, *F*_(2, 12)_ = 4.562, *p < 0.05). **d** Representative Western blots showing Cx32, α-syn and GAPDH protein levels following SB203580 treatment. **e** Densitometric analysis of Cx32 protein expression in differentiated SH-SY5Y cells exposed to anisomycin for 24 h, (*n* = 5, one-way ANOVA followed by Tukey’s post hoc test for multiple comparisons, n.s; no significance, *F*_(3, 11)_ = 4.553, *p < 0.05). **f** qRT-PCR analysis of Cx32 mRNA in differentiated SH-SY5Y cells treated with increasing anisomycin concentrations and untreated cells as controls (*n* = 3, one-way ANOVA followed by Tukey’s post hoc test for multiple comparisons, n.s; no significance, *F*_(3, 8)_ = 3.998, *p < 0.05). **g** Densitometric analysis of oα-syn uptake following anisomycin treatment for 24 h (*n* = 4, one-way ANOVA followed by Tukey’s post hoc test for multiple comparisons, n.s; no significance, *F*_(3, 12)_ = 5.327, *p < 0.05). **h** Representative Western blots showing Cx32, α-syn and GAPDH protein levels following anisomycin treatment. **i**, **j** Western blot analysis of oα-syn uptake in SH-SY5Y cells expressing Cx32-mCherry (*n* = 6) or mCherry (*n* = 4) incubated at 37 °C or 4 °C (one-way ANOVA followed by Tukey’s post hoc test for multiple comparisons, n.s; no significance, *F*_(3, 16)_ = 83.02, ***p < 0.001, ****p < 0.0001). **k** Densitometric analysis of **l** Western blots of oAβ uptake in differentiated SH-SY5Y cells expressing mCherry or Cx32-mCherry (*n* = 4, unpaired, two-tailed *t* test, *t*_6_ = 8.493, ***p = 0.0001). **m** Confocal image analysis of differentiated SH-SY5Y cells expressing mCherry or Cx32-mCherry (red) labeled with Aβ (6E10, green) and then stained with Hoechst (blue); scale bar represents 200 µm. **n** Fluorescence intensity quantification of oAβ uptake in differentiated SH-SY5Y cells expressing mCherry or Cx32-mCherry by confocal microscopy (*n* = 3 independent experiments and each representing the average of three different measurements, unpaired, two-tailed *t* test, *t*_4_ = 11.86 ***p < 0.001)

To further demonstrate a direct link between Cx32 expression and oα-syn uptake in differentiated human neuronal SH-SY5Y cells, we investigated the impact of inhibiting the p38 MAPK pathway, which negatively regulates Cx32 protein turnover, on oα-syn uptake [39]. We incubated differentiated SH-SY5Y cells with the potent p38 MAPK inhibitor SB203580 (10 and 25 μM), together with oα-syn for 24 h. As expected, we observed a concentration-dependent increase in Cx32 protein expression and this effect led to a decrease in Cx32 mRNA (Fig. 2a, b). Correspondingly, the increase in Cx32 protein expression induced by SB203580 treatment correlated with a significant increase in oα-syn uptake that was concentration dependent (Fig. 2c, d). In contrast, cells exposed to the p38 MAPK activator anisomycin (1, 3, and 5 µM), which promotes Cx32 degradation [39], showed a significant decrease in Cx32 protein expression and promoted a significant upregulation of Cx32 mRNA (Fig. 2e, f). Concomitant with the results above, the observed reduction in Cx32 expression correlated with a significant decrease in oα-syn uptake, emphasizing a direct link between Cx32 expression and oα-syn uptake (Fig. 2g, h).

### Cx32-mediated protein uptake is energy dependent and preferential for oα-syn assemblies

Next, we investigated whether Cx32-mediated oα-syn uptake is mediated by an energy-dependent mechanism rather than via channel diffusion. We incubated Cx32-mCherry and mCherry control cells with oα-syn assemblies at 4 °C or 37 °C for 6 h. As expected, we observed a greater uptake of oα-syn in cells expressing Cx32-mCherry than its wild-type counterpart (mCherry) under normal culture conditions (37 °C) (Fig. 2i). However, reduced temperatures (4 °C) significantly reduced oα-syn uptake in both cell types, indicating an energy-dependent mechanism required for oα-syn uptake (Fig. 2i, j). Given the accumulation of protein oligomers in other neurodegenerative conditions including AD [48], we next assessed whether Cx32 mediates the uptake of oligomers composed of Aβ, one of the pathological hallmarks of AD [34]. Similar to our oα-syn uptake conditions, we incubated differentiated SH-SY5Y cells expressing Cx32 or mCherry proteins alone for 24 h then assessed oAβ uptake by immunocytochemistry and Western blot analysis. We observed a significant increase in the uptake of oAβ in cells expressing Cx32 compared to controls by both Western blot analysis and immunocytochemistry (Fig. 2k–n). However, the differential uptake of oAβ via Cx32 compared to controls was not as effective as that for oα-syn assemblies (compare Figs. 1j and 2k), suggesting that, in addition to its oligomeric state, the underlying protein sequence may play a role in this process.

### Cx32 expression increases oα-syn cell-to-cell transfer

**Fig. 3:**
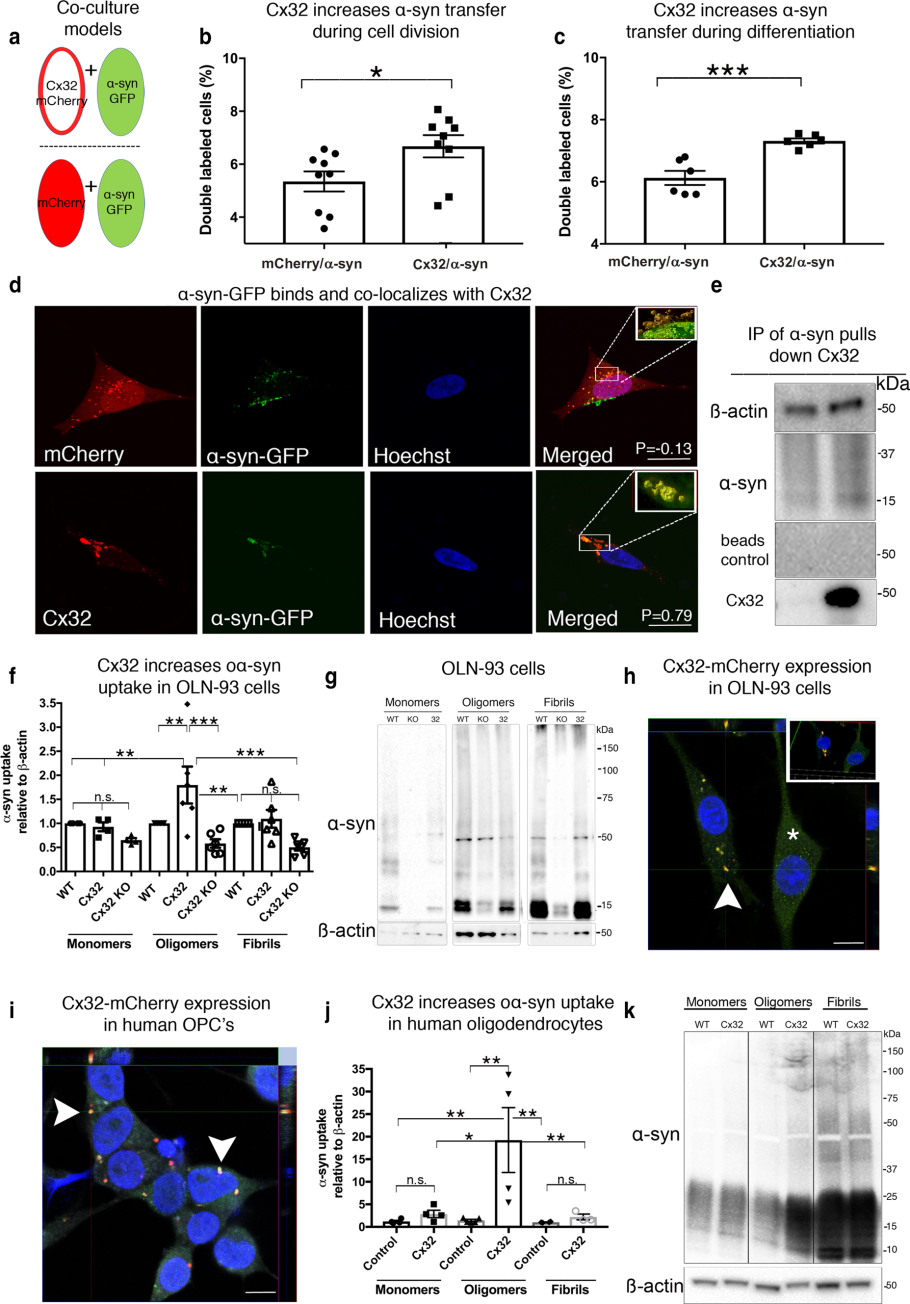
Cx32 interacts with oα-syn and facilitates uptake and transfer in neurons and oligodendrocytes. **a** Schematic representation of the co-culture donor and recipient SH-SY5Y models designed to quantify α-syn-GFP transfer as donor cells (green) and recipient cells shown in red by open (Cx32-mCherry) or closed circle (mCherry), depicting the relative expression of the plasmids. **b** Flow cytometry measurement of α-syn-GFP transfer in undifferentiated conditions (*n* = 9 independent experiments and each representing the average of triplicate measurements, unpaired, two-tailed *t* test, *t*_16_ = 2.35, *p < 0.031). **c** Flow cytometry analysis of α-syn-GFP transfer in a differentiated state (*n* = 6 independent experiments and each representing the average of duplicate measurements, unpaired, two-tailed *t* test, *t*_10_ = 4.03, ***p < 0.001). **d** Confocal image analysis of recipient cells (mCherry and Cx32-mCherry) containing α-syn-GFP oligomers sorted by FACS and analyzed by Huygens Pro (top 3D insert), demonstrating colocalization between Cx32 and α-syn-GFP as indicated by the Pearson correlation coefficient (P) of 0.79 compared to -0.13 for α-syn-GFP and mCherry; scale bars represent 20 µm. **e** Immunoprecipitation (IP) of oα-syn followed by Western blot analysis with mCherry and α-syn antibodies identifies an interaction between oα -syn and Cx32 **f** Densitometric analysis of **g** representative Western blots of α-syn uptake in monomeric, oligomeric or fibrillar assemblies in differentiated WT rat oligodendrocytes (OLN-93) or oligodendrocytes expressing Cx32-mCherry or cells lacking Cx32 (Cx32-KO) (*n* = 6, two-way ANOVA followed by Tukey’s post hoc test for multiple comparisons, n.s; no significance, *F*_(8, 24)_ = 6.13, *p < 0.05, **p < 0.01 ***p < 0.001). **h** Immunocytochemistry of OLN-93 cells overexpressing Cx32-mCherry showing the typical Cx32-mCherry plaques at the cell membrane (red, arrowheads), or cell soma (*) co-labeled with Cx32 antibody (green) and Hoechst (blue), scale bars represent 10 µm. Top insert represents a 3D image demonstrating the Cx32 plaque at the cellular membrane (arrow) and cytoplasm (*) **i** Immunocytochemistry and orthogonal view of human oligodendrocyte precursor cells (OPCs) overexpressing Cx32-mCherry (at the cell membrane, arrowheads) stained with Hoechst (blue) and CNPase (gray); scale bars represent 10 µm. **j** Densitometric analysis of **k** representative Western blot of α-syn uptake (monomeric, oligomeric or fibrillar) in differentiated human WT oligodendrocytes or oligodendrocytes overexpressing Cx32-mCherry (*n* = 4, two-way ANOVA followed by Tukey’s post hoc test for multiple comparisons, n.s; no significance, *F*(_5,15_) = 5.914, *p < 0.05, **p < 0.01)

In a previous study, we generated a co-culture model system under proliferating conditions to monitor and quantify the transfer of human α-syn-GFP from donor to recipient cells that was suitable for flow cytometry and high content screening [43]. Using a similar approach (Fig. 3a), we assessed whether proliferating human SH-SY5Y cells overexpressing Cx32-mCherry (as recipient cells) efficiently internalized α-syn-GFP derived from donor cells compared to cells expressing untagged mCherry proteins. Following 5 days of co-culture, we quantified a higher percentage of Cx32-mCherry cells containing α-syn-GFP puncta compared to control cells expressing untagged mCherry proteins (Fig. 3b). We next co-cultured donor and recipient cells under differentiation conditions to promote a mature neuron-like phenotype and enhance neuronal connectivity [2] (Suppl. Figure S2a, b, Online Resource 5). Consistent with our results above, we observed a higher percentage of Cx32-mCherry cells containing α-syn-GFP puncta compared to control cells expressing mCherry proteins alone under differentiating conditions (Fig. 3c). Although a higher trend for α-syn-GFP transfer in cells under differentiation was observed compared to proliferating conditions, this difference was not statistically significant. To further assess the role of neuronal connectivity in the transfer of α-syn, we co-cultured donor and recipient cells in separate compartments to prevent cellular connectivity but allowing co-cultured cells to share the same medium (Suppl. Figure S2c, Online Resource 5). Consistent with the results above, neuronal connectivity increased the transfer of α-syn-GFP to cells expressing Cx32-mCherry compared to mCherry controls. However, blocking neuronal connectivity prevented α-syn transfer even though the presence of oα-syn within the media could be demonstrated [43]. Collectively, our results indicate that neuronal connectivity plays a major role in the transfer of oα-syn, suggesting that Cx32 expression increases oα-syn uptake in a manner enhanced by neuronal connectivity.

### oα-Syn binds and colocalizes with Cx32 during cellular uptake

To validate oα-syn transfer to recipient cells during our co-culture conditions, we sorted double-labeled cells expressing either Cx32-mCherry or mCherry containing α-syn-GFP puncta using fluorescence-activated cell sorting (FACS). Using confocal microscopy, we visualized the presence of α-syn-GFP puncta within the cell soma and around the cell membrane of both Cx32-mCherry and mCherry recipient cells (Fig. 3d, Suppl. Figure S2d, Online Resource 5). We then evaluated the extent of colocalization between α-syn-GFP and Cx32-mCherry fluorophores or α-syn-GFP and mCherry by calculating the Pearson correlation coefficient (*P*). Our results point to a direct protein–protein interaction between oα-syn and Cx32 during cellular uptake that is not evident in cells expressing mCherry proteins alone (Fig. 3d, Suppl. Figure S2d, Online Resource 5). To further assess a direct interaction between Cx32 and oα-syn, we incubated Cx32-mCherry or mCherry cells with oα-syn for 24 h followed by immunoprecipitation (IP) of oα-syn assemblies using a pan-specific antibody that does not interfere with the N-terminal region of α-syn required for membrane interaction [37] (epitope 117-125). Given the observed interaction between Cx32 and oα-syn by confocal microscopy, we identified the presence of Cx32-mCherry but not of untagged mCherry proteins following IP of oα-syn (Fig. 3e). Moreover, the presence of beads with IgG in our IP conditions failed to pull down α-syn or Cx32 within the samples tested, confirming a direct interaction between Cx32 and oα-syn during cellular uptake.

### Cx32 interacts with oα-syn and facilitates uptake in oligodendrocytes

We next determined whether Cx32 also plays a role in the uptake of oα-syn in oligodendrocytes, the cell type associated with canonical Cx32 expression in the central nervous system [40]. Using the rat oligodendrocyte cell line OLN-93 [45], we generated Cx32-KO cells using CRISPR/Cas9 or overexpressed Cx32-mCherry. We then differentiated these cells by serum deprivation as previously reported [45], followed by incubation for 24 h with α-syn monomers, oligomers or fibrillar assemblies. Consistent with our Cx32 neuronal model system, we observed a significant increase in the uptake of oα-syn assemblies compared to monomers and fibrillar assemblies in Cx32-mCherry OLN-93 cells (Fig. 3f, g). Moreover, OLN-93 cells lacking Cx32 (Cx32-KO) showed a decrease in oα-syn uptake compared to control conditions and although this was not statistically significant, it validates the involvement of other cellular mechanisms in this process (Fig. 3f, g). It is worth noting that whereas Cx32 in SH-SY5Y cells was observed at the cellular membrane (Fig. 1l), Cx32-mCherry in OLN-93 oligodendrocytes localized primarily to the cell soma (Fig. 3h, asterisk). However, in some instances, we found that Cx32 was properly localized to the cellular membrane, forming the typical Cx32 plaques (Fig. 3h, arrowheads).

To further validate the involvement of Cx32 in the uptake of oα-syn in oligodendrocytes, we transfected human oligodendrocyte precursor cells (OPCs) derived from induced pluripotent stem cells (iPS) with Cx32-mCherry or untagged mCherry proteins. We observed Cx32-mCherry in differentiated human OPCs (2′,3′-Cyclic-nucleotide 3′-phosphodiesterase, CNPase+) localized to the cellular membrane, where it remained throughout the differentiation process (Fig. 3i). In contrast, untagged mCherry expression was observed throughout the entire cytoplasm (data not shown). We next differentiated OPCs for 30 days and used the expression of the myelin basic protein (MBP) by immunocytochemistry as a marker for cell maturity (Suppl. Figure S3a, b, Online Resource 6). Subsequently, we incubated differentiated OPCs with α-syn monomers, oligomers or fibrillar assemblies for 24 h. Using Western blot analysis, we found that expression of Cx32-mCherry in differentiated oligodendrocytes (MBP+) significantly increased the uptake of oα-syn assemblies compared to monomeric or fibrillar assemblies (Fig. 3j, k). To validate a direct interaction between oα-syn and Cx32 in human oligodendrocytes, we next treated differentiated OPCs (CNPase+) overexpressing Cx32-mCherry with ATTO-488-labeled oα-syn for 24 h. Similar to the results in our neuronal culture model, we observed a remarkable colocalization between Cx32 and oα-syn-ATT0-488 proteins in human oligodendrocytes (Suppl. Figure S3c. Online Resource 6). We next treated OPCs expressing Cx32-mCherry cells with oα-syn followed by IP of oα-syn as described above, and identified the presence of Cx32 within the immunoprecipitated samples by Western blot analysis (Suppl. Figure S3d, Online Resource 6). Collectively, our results confirm that Cx32 interacts with oα-syn and facilitates its internalization in neurons and oligodendrocytes.

### Cx32 peptide mimetic inhibitors reduce the uptake of oα-syn assemblies

**Fig. 4:**
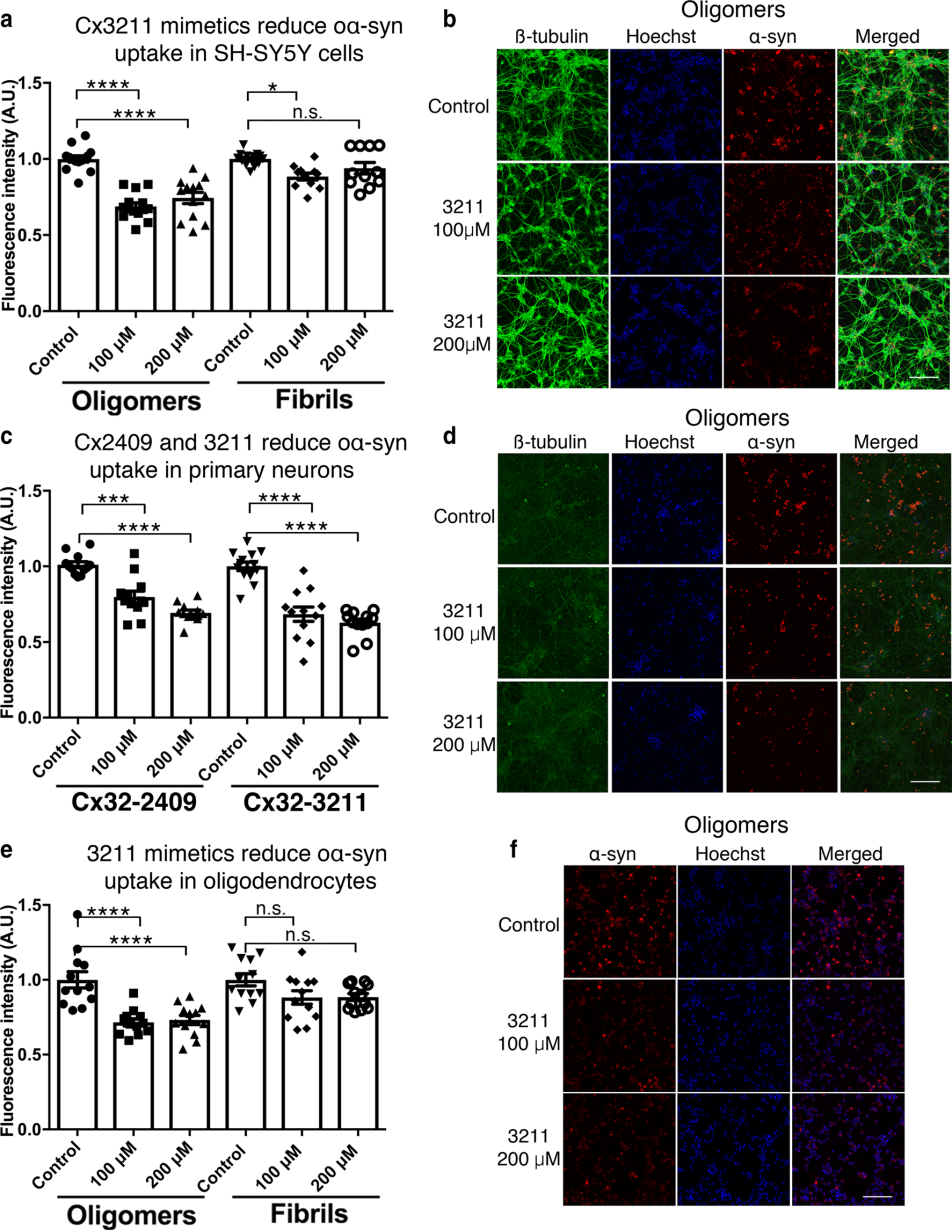
Cx32 peptide mimetics block oα-syn uptake. **a** Fluorescence intensity measurements of α-syn uptake in differentiated SH-SY5Y cells exposed to oα-syn or fibrillar assemblies in the presence of Gap3211 peptide mimetics (*n* = 14 in 4 independent experiments, one-way ANOVA followed by Tukey’s post hoc test for multiple comparisons, n.s; no significance, *F*_(5, 70)_ = 26.33, *p < 0.01, ****p < 0.0001). **b** Representative micrographs of differentiated human SH-SY5Y cells co-labeled with β3-tubulin (green), α-syn (ATTO-550) and Hoechst (blue) visualized under confocal microscopy. **c** Fluorescence intensity measurements of α-syn-ATTO-550 uptake in primary cortical neurons (DIV 10) exposed to oα-syn assemblies in the presence of Gap2409 and Gap3211 peptide mimetics, (*n* = 12 in 4 independent experiments, one-way ANOVA followed by Tukey’s post hoc test for multiple comparisons, n.s; no significance, *F*_(5, 67)_ = 28.93, ***p < 0.001, ****p < 0.0001). **d** Representative micrographs of primary rat cortical neurons co-labeled with β3-tubulin (green), α-syn (ATTO-550) and Hoechst (blue) visualized under confocal microscopy. **e** Fluorescence intensity measurements of α-syn-ATTO-550 uptake in differentiated OLN-93 oligodendrocyte cells exposed to oα-syn or fibrillar assemblies in the presence of Gap3211 peptide mimetics (*n* = 12 in 4 independent experiments, one-way ANOVA followed by Tukey’s post hoc test for multiple comparisons, n.s; no significance, *F*_(5, 66)_ = 10.61, ****p < 0.0001). **f** Representative micrographs of differentiated OLN-93 oligodendrocytes co-labeled with α-syn (ATTO-550) and Hoechst (blue) visualized under confocal microscopy. Scale bars represent 100 µm

We next explored whether pharmacological strategies targeting Cx32 activity inhibit oα-syn uptake. We exposed differentiated wild-type SH-SY5Y cells to oα-syn or fibrillar assemblies tagged with ATTO-550 [45], in the presence of specific Cx32 peptide mimetic sequences (Gap3211 and Gap2409) known to bind and block Cx32 hemichannel activity [9, 13]. Gap3211 binds to the first extracellular loop [amino acid (AA) 52–63], whereas Gap2409 binds to the inner cytoplasmic transmembrane loop (AA100–110) of Cx32. Treatment of differentiated SH-SY5Y cells with oα-syn assemblies in the presence of Gap3211 (Fig. 4a, b) or Gap2409 (Suppl. Figure S4a, c, Online Resource 7) for 24 h significantly reduced oα-syn uptake. This treatment, however, had a limited effect on fibrillar α-syn uptake (Fig. 4a, Suppl. Figure S4a, e, Online Resource 7). Given the high sequence similarity of Cx32 between humans and rodents, we next exposed primary rat cortical neurons (DIV 10) to oα-syn assemblies in the presence of Gap3211 or Gap2409. Consistent with our differentiated human neuronal SH-SY5Y results, we observed a significant reduction in oα-syn uptake following Gap3211 or Gap2409 peptide mimetic treatment on primary neurons (Fig. 4c, d). Similarly, OLN-93 oligodendrocytes exposed to oα-syn or fibrillar assemblies in the presence of Gap3211 or Gap2409 showed a marked decrease in oα-syn uptake (Fig. 4e, f, Suppl. Figure S4b, d); however, this effect did not translate to fibrillar assemblies (Fig. 4e, f Suppl. Figure S4b, f, Online Resource 7).

To verify that inhibition of oα-syn is dependent on the sequence of Cx32, we next treated differentiated human neuronal SH-SY5Y cells with Cx32 scrambled mimetic sequences (SC) of both GAP3211-SC (Suppl. Figure S5a, Online Resource 8) and 2409-SC (data not shown). In contrast to functional Cx32 sequences, we observed no inhibition of oα-syn or fibrillar uptake using Cx32-SC (Suppl. Figure S5a, Online Resource 8). We next assessed whether functional mimetic sequences targeting Cx43 blocked oα-syn or fibrillar uptake. We treated cells with peptides targeting the first extracellular loop of Cx43 (Gap2605, AA64–76), but no effect on oligomeric or fibrillar α-syn assemblies could be detected (Suppl. Figure S5b, Online Resource 8). These results indicate that blockade of Cx32 using peptide mimetic sequences partially impedes the uptake of oα-syn, but this treatment has limited effect on fibrillar α-syn assemblies.

### Pharmacological gap junction inhibitors reduce α-syn uptake

**Fig. 5:**
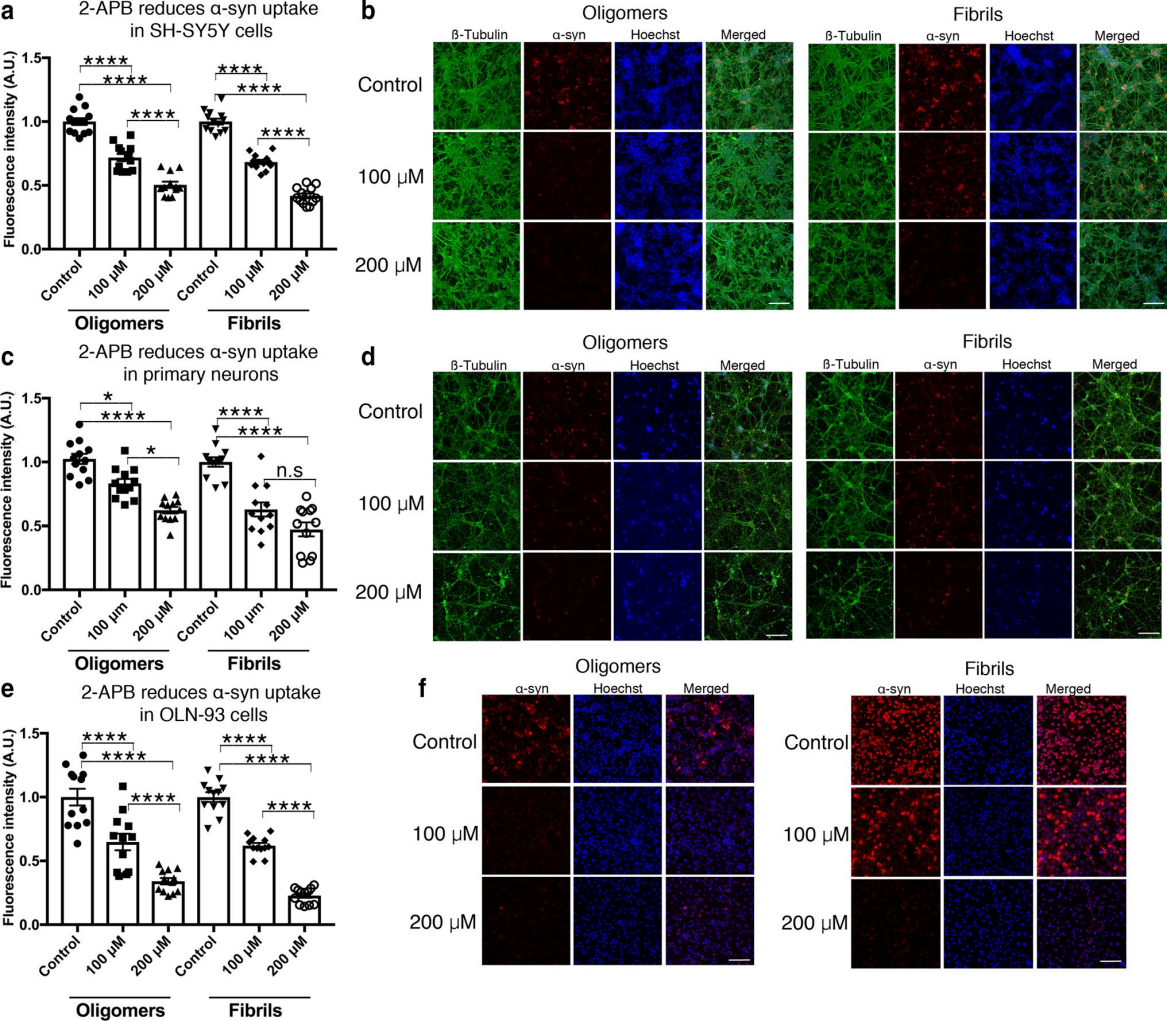
Selective gap junction inhibitor 2-APB blocks the uptake of oligomeric and fibrillar α-syn assemblies. **a** Fluorescence intensity measurements of oα-syn or fibrillar α-syn uptake in differentiated human SH-SY5Y cells in the presence of 2-APB, (*n* = 12 in four independent experiments, one-way ANOVA followed by Tukey’s post hoc test for multiple comparisons, *F*_(5, 70)_ = 108.6, ****p < 0.0001). **b** Representative micrographs of differentiated human SH-SY5Y cells co-labeled with β3-tubulin (green), α-syn (ATTO-550) and Hoechst (blue) visualized using confocal microscopy. **c** Fluorescence intensity measurements of oα-syn or fibrillar α-syn uptake in primary rat cortical neurons (DIV 10) in the presence of 2-APB (*n* = 12 in 4 independent experiments, one-way ANOVA followed by Tukey’s post hoc test for multiple comparisons, n.s; no significance, *F*_(5, 66)_ = 27.88, *p < 0.05, ****p < 0.0001). **d** Representative micrographs of primary cortical neurons co-labeled with β3-tubulin (green), α-syn (ATTO-550) and Hoechst (blue) visualized using confocal microscopy **e** Fluorescence intensity measurements of oα-syn and fibrillar uptake in differentiated OLN-93 oligodendrocytes in the presence of 2-APB (*n* = 12 of 4 independent experiments, one-way ANOVA followed by Tukey’s post hoc test for multiple comparisons, *F*_(5, 70)_ = 54.37, ****p < 0.0001). **f** Representative micrographs of differentiated OLN-93 oligodendrocytes co-labeled with α-syn (ATTO-550) and Hoechst (blue) visualized using confocal microscopy. Scale bars represent 100 µm

To further assess the potential of Cx32 as a suitable target for pharmacological intervention to block α-syn uptake, we next tested the widely used pan-connexin gap junction inhibitor carbenoxolone (CBX) [21]. Treatment of differentiated neuronal SH-SY5Y cells with increasing concentrations of CBX (50 and 100 µM) significantly blocked oα-syn and fibrillar uptake in a concentration-dependent manner (Suppl. Figure S6a, b, Online Resource 9). Similarly, treatment of oligodendrocytes (OLN-93) with CBX led to a significant decrease in oα-syn and fibrillar uptake that was concentration dependent (Suppl. Figure S6c, d, Online Resource 9). Given the non-specific inhibitory profile of CBX to different Cx proteins, we next assessed the effect of mefloquine (MQ), a selective inhibitor that targets gap junctions composed primarily of Cx26, Cx32 and/or Cx43 [21]. In our culture conditions, MQ (25 and 50 µM) significantly blocked the uptake of oα-syn and fibrillar assemblies in human differentiated SH-SY5Y neuronal cells, primary cortical neurons (DIV 10) and differentiated OLN-93 oligodendrocytes in a concentration-dependent manner (Suppl. Figure S7a–f, Online Resource 10). Finally, we tested the inhibitory effect of 2-aminoethoxydiphenyl borate (2-APB), a highly selective gap junction inhibitor known to primarily target Cx26 and Cx32 [63]. Treatment of cells with 2-APB (100 and 200 µM) significantly blocked the uptake of oα-syn and fibrillar assemblies in human SH-SY5Y neuronal cells (Fig. 5a, b), primary cortical neurons (DIV 10) (Fig. 5c, d) and OLN-93 oligodendrocytes in a concentration-dependent manner (Fig. 5e, f).

### α-Syn expression differentially regulates Cx32 in neurons and oligodendrocytes

**Fig. 6:**
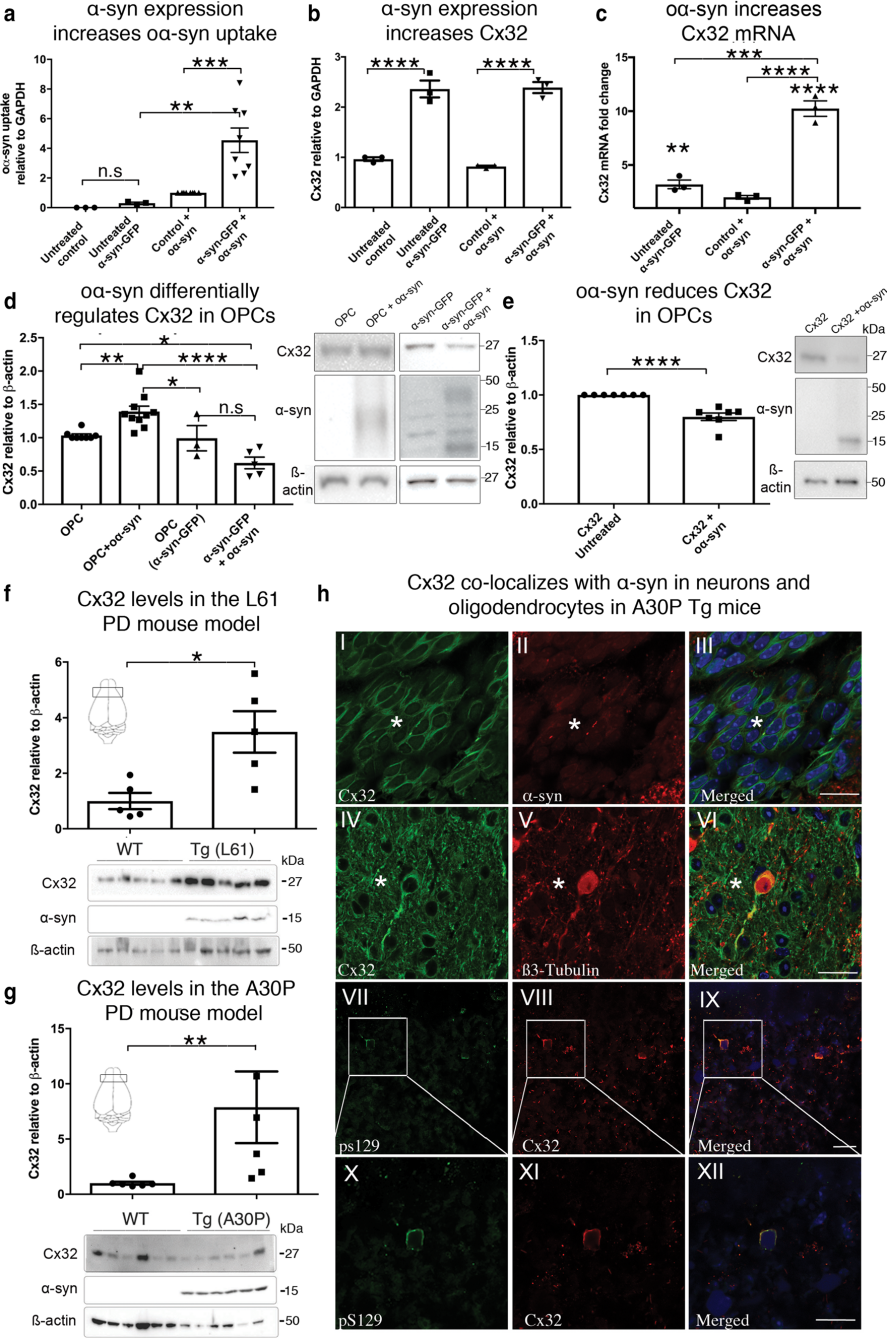
Human α-syn expression promotes Cx32 upregulation in vitro and in vivo in animal models of PD. **a** Densitometric analysis of oα-syn oligomer uptake in control SH-SY5Y cells (*n* = 3) or cells expressing α-syn-GFP (*n* = 8, one-way ANOVA followed by Tukey’s post hoc test for multiple comparisons, n.s; no significance, *F*_(3,18)_ = 12.33, **p < 0.01, ***p < 0.001). **b** Protein analysis of Cx32 expression in control and α-syn-GFP-expressing cells before and after oα-syn treatment (*n* = 3, one-way ANOVA followed by Tukey’s post hoc test for multiple comparisons, *F*_(3, 8)_ = 70.33, ****p < 0.0001). **c** mRNA analysis of Cx32 in control and α-syn-GFP-expressing cells before and after oα-syn treatment (*n* = 3, one-way ANOVA followed by Tukey’s post hoc test for multiple comparisons, *F*_(3, 8)_ = 54.24, **p < 0.01, ***p < 0.001, ****p < 0.0001). **d** Densitometric analysis and Western blot of control WT OPCs (*n* = 8) and OPCs exposed to α-syn assemblies (*n* = 10) or OPCs expressing α-syn-GFP with or without oα-syn treatment (*n* = 3 and 5, respectively, one-way ANOVA followed by Tukey’s post hoc test for multiple comparisons, n.s; no significance, *F*_(2, 22)_ = 14.94, *p < 0.05, **p < 0.01, ****p < 0.0001). **e** Densitometric analysis of OPCs expressing Cx32-mCherry exposed to oα-syn assemblies (*n* = 7, unpaired, two-tailed *t* test, t_8_ = 5.836, ****p < 0.0001). **f** Densitometric analysis of wild-type (non-Tg) and transgenic (L61) mice overexpressing human wild-type α-syn (Mann–Whitney *U *= 2, *n*_1_ = *n*_2_ = 5 *p < 0.05) or **g** α-syn harboring the familial A30P mutation (Mann–Whitney *U *= 1,* n*_1_ = *n*_2_ = 6 *p < 0.05). Schematic representations within the figures show the location of the brain region selected for analysis. **h** Immunohistochemistry of A30P mouse tissue sections co-labeled with the Cx32 antibody and human pan-specific α-syn antibody shows colocalization between α-syn and Cx32 in oligodendrocytes (I–III, *), between Cx32 and the neuron-specific β3-tubulin antibody (IV–VI, *) or between Cx32 and the phospho-specific pS129-specific α-syn antibody (VII–XII). The boxed area shows a high magnification of VII–IX (X–XII). Scale bars represent 20 µm

Having established that Cx32 expression preferentially modulates oα-syn uptake in neurons and oligodendrocytes, we next assessed the effect of oα-syn uptake in differentiated SH-SY5Y cells overexpressing human α-syn-GFP, a useful cellular system to model PD in vitro [26]. We treated differentiated SH-SY5Y cells overexpressing human α-syn-GFP with exogenously generated oα-syn assemblies for 24 h, then measured protein uptake by Western blot analysis. We observed a significant increase in the uptake of oα-syn in cells expressing α-syn-GFP compared to controls (Fig. 6a). Importantly, this effect was not due to the proteolytic degradation of the endogenous α-syn-GFP construct, which remained fully intact (~ 40 kDa) throughout the experiment (Suppl. Figure S8a, Online Resource 11). Prompted by the observed increase in oα-syn uptake in our in vitro cellular model of PD, we next examined the levels of Cx32 protein and mRNA before and after oα-syn treatment. We found that Cx32 protein levels increased upon expression of α-syn-GFP compared to controls (Fig. 6b), with no further detectable increase following exogenous oα-syn treatment. However, an increase in Cx32 mRNA following exogenous oα-syn treatment compared to that of the untreated α-syn-GFP cells was observed, an effect that was more pronounced in the treated α-syn-GFP expressing cells than in wild-type-treated controls (Fig. 6c).

Next, we investigated whether Cx32 upregulation correlates with the levels of α-syn expression. We, therefore, separated SH-SY5Y cells expressing low and high levels of α-syn-GFP using FACS (Suppl. Figure S8b, Online Resource 11). We then induced neuronal differentiation as previously reported [2], and assessed the levels of Cx32 expression by Western blot and qRT-PCR. Interestingly, we identified an increase in Cx32 protein upregulation that associated with the levels of α-syn-GFP expression, both at the protein and mRNA level (Suppl. Figure S8c, d, Online Resource 11), demonstrating a direct correlation between Cx32 upregulation and α-syn expression, (Suppl. Figure S8e, Online Resource 11). Collectively, our results suggest that expression of human α-syn or exposure to oα-syn assemblies promotes Cx32 upregulation in differentiated human neuronal SH-SY5Y cells.

We next investigated whether exogenous oα-syn treatment or expression of α-syn-GFP in human oligodendrocytes would increase Cx32 expression. We exposed differentiated wild-type OPCs (CNPase +) to oα-syn assemblies for 24 h then measured Cx32 protein expression. As expected, we observed a significant increase in Cx32 upregulation, albeit to a lesser extent than that observed in neuronal SH-SY5Y cells (Fig. 6d). However, in contrast to the neuronal SH-SY5Y model, we identified no differences in Cx32 protein expression between wild-type OPCs and OPCs overexpressing human α-syn-GFP (Fig. 6d). In fact, relative to wild-type OPC-treated cells, OPC-α-syn-GFP cells exposed to exogenous oα-syn assemblies showed a significant decrease in Cx32 expression. However, although a trend for a reduction in Cx32 expression was observed following incubation of OPC’s expressing α-syn-GFP with oα-syn assemblies compared to untreated α-syn-GFP cells, this effect was not statistically significant (Fig. 6d). We then asked whether OPCs overexpressing Cx32 would show a decrease in Cx32 following oα-syn treatment. We incubated OPCs overexpressing Cx32-mCherry with oα-syn proteins for 24 h then measured Cx32 protein levels by Western blot analysis. We observed a significant decrease in endogenous Cx32 protein expression in cells incubated with oα-syn compared to untreated OPC-Cx32 cells (Fig. 6e). These results suggest that, compared to neuronal cells, human oligodendrocytes differentially regulate Cx32 expression following α-syn expression or exposure to oα-syn assemblies.

### α-Syn accumulation increases Cx32 in Tg models of PD and MSA

To assess a possible connection between α-syn and Cx32 in vivo, we analyzed adult and aged cortical brain homogenate samples from Tg mice overexpressing human wild-type α-syn (Line 61, 6 months) or mutant α-syn harboring the familial A30P mutation (18 months), both driven by the Thy-1 promoter (Suppl. Table S2 [23, 46], Online Resource 2). Compared to respective non-transgenic (non-Tg) age-matched controls, we observed a significant upregulation in Cx32 protein expression in L61 (Fig. 6f) and A30P Tg mice (Fig. 6g). The upregulation of monomeric Cx32 was corroborated using a different immunological probe targeting the C-terminal region of the Cx32 molecule (Suppl. Figure S8f, g, Online Resource 11). We then analyzed the levels of Cx32 mRNA in respective age-matched control mice and L61 and A30P cohorts. While we observed no statistically significant differences between non-Tg and Tg models (L61 and A30P), a trend of increased Cx32 mRNA levels in both PD models was noted (Suppl. Figure S8 h, Online Resource 11). To further assess whether Cx32 colocalizes with α-syn in situ, we performed immunohistochemistry on tissue sections from the A30P mice (Fig. 6h). We observed a clear colocalization between Cx32 and human A30P-α-syn that, in some instances, appeared within the membrane of cells of the oligodendrocyte lineage, as indicated by the morphological pattern of Cx32 labeling within oligodendrocytes (Fig. 6h, I–III). To further validate the presence of α-syn within cells of the oligodendrocyte lineage, we performed immunohistochemistry on adjacent tissue sections using the oligodendrocyte-specific marker CNPase and observed the presence of α-syn within CNPase+ cells [Suppl. Figure S9a (I–IV), Online Resource 12]. Moreover, we observed a clear colocalization between Cx32 and the neuronal-specific protein β3-tubulin, confirming the presence of Cx32 proteins within neuronal cell types (Fig. 6h, IV–VI). Finally, we immunolabeled tissue sections with a highly specific α-syn antibody that labels α-syn phosphorylated at serine 129 (pS129) and does not cross-react with a similar epitope in the neurofilament light chain protein (NFL [51]). Our qualitative observations revealed a clear colocalization between Cx32 and pS129 in neuronal cells (Fig. 6h, VII–XII) and oligodendrocytes co-labeled with CNPase and pS129 [Suppl. Figure S9a (V–VIII), Online Resource 12], corroborating the direct interaction between Cx32 and α-syn in neuronal and oligodendrocyte cell types in the Tg A30P mouse model of PD.

**Fig. 7:**
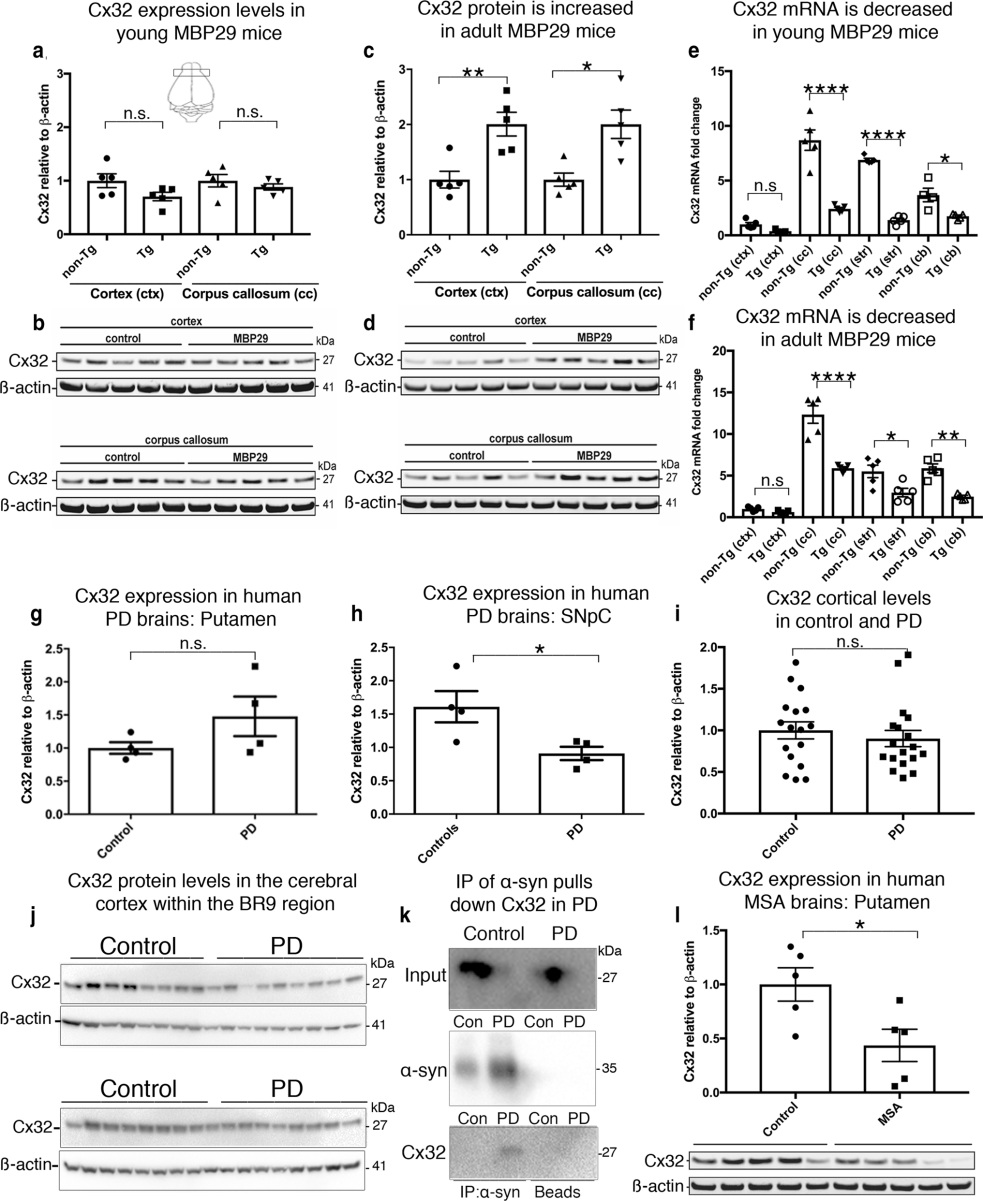
α-syn promotes Cx32 upregulation in a mouse model of MSA (MBP29) and shows a direct interaction with Cx32 in human PD brains. **a** Densitometric analysis of **b** a representative Western blot of Cx32 in young (3 weeks) non-Tg and Tg MBP29 cohorts from regions of the cortex and corpus callosum (*n* = 5, one-way ANOVA followed by Tukey’s post hoc test for multiple comparisons, n.s; no significance, *F*_(3, 16)_ = 1.968). Schematic representation within figure a shows the location of the brain region selected for analysis. **c** Densitometric analysis of **d** representative Western blot of Cx32 in adult (3 months) non-Tg and Tg MBP29 cohorts from regions of the cortex and corpus callosum (*n* = 5, one-way ANOVA followed by Tukey’s post hoc test for multiple comparisons, *F*_(3, 16)_ = 8.991, *p < 0.05, **p < 0.01). **e** Cx32 mRNA analysis *(2*^−*ddct*^) of young non-Tg (*n* = 5) and Tg MBP29 cohorts (*n* = 5, one-way ANOVA followed by Tukey’s post hoc test for multiple comparisons, n.s; no significance, *F*_(7, 31)_ = 57.88, *p < 0.05, ****p < 0.0001). **f** Cx32 mRNA analysis *(2*^−*ddct*^) of adult non-Tg (*n* = 5) and Tg MBP29 cohorts (*n* = 5, one-way ANOVA followed by Tukey’s post hoc test for multiple comparisons, n.s; no significance, one-way ANOVA followed by Tukey’s post hoc test for multiple comparisons, n.s; no significance, *F*_(7, 32)_ = 50.74 *p < 0.05, **p < 0.01, ****p < 0.0001). **g** Densitometric analysis of Western blot of Cx32 in human brain homogenate samples from the putamen region of control (*n* = 4) and PD cases (*n* = 4, unpaired, two-tailed *t* test, n.s; no significance, *t*_6_ = 1.56). **h** Densitometric analysis of Western blot of Cx32 in human brain homogenate samples from the SNpc isolated from human PD cases (*n* = 4) and age-matched controls (*n* = 4, unpaired, two-tailed *t* test, *t*_6_ = 2.753 *p < 0.05). **i** Densitometric analysis of Cx32 **j** from the cortex within the BR9 region of control and PD cases. **k** Representative Western blot of α-syn IP from the human putamen isolated from controls and PD cases demonstrates a direct interaction between Cx32 and human α-syn in PD (2 out of 4) cases but not in age-matched controls (*n* = 4). **l** Western blot analysis of Cx32 protein levels within the putamen of control (*n* = 5) or MSA cases (*n* = 5, unpaired, two-tailed *t* test, n.s; no significance, *t*_8_ = 2.627, *p < 0.05)

We then investigated the relationship between Cx32 and α-syn in the MBP29 mouse model of MSA, which overexpresses human wild-type α-syn in oligodendrocytes driven by the MBP promoter [62]. We prepared brain homogenate samples from the cortex (ctx) and corpus callosum (cc) of young (3 weeks) and adult cohorts (3 months) followed by Western blot analysis. We observed no statistically significant differences in the Cx32 profile between young age-matched controls and Tg MBP29 mice (Fig. 7a, b). Importantly, and consistent with the accumulation of α-syn deposits within these mice over time, we observed a significant age-dependent increase in Cx32 protein levels in Tg cohorts compared to age-matched controls (3 months, Fig. 7c, d) as well as young MBP29 Tg mice (Suppl. Figure S9b, Online Resource 12). These results are similar to those in the mouse PD cohorts; however, a significant decrease in Cx32 mRNA in both young and adult Tg MBP29 cohorts compared to non-Tg age-matched controls within the cortex and corpus callosum was clearly observed (Fig. 7e, f). These results are consistent with our in vitro oligodendrocyte results pointing to an overall attempt to downregulate Cx32 in oligodendrocytes following oα-syn expression, indicating that Cx32 expression in oligodendrocytes is differentially regulated compared to its neuronal counterpart.

### Cx32 interacts with α-syn in PD brains

To further assess the relationship between Cx32 and α-syn in human PD brains, we analyzed the putamen and substantia nigra pars compacta (SNpc) from four neuropathologically diagnosed PD cases and four age-matched controls (Suppl. Table S3, Online Resource 3). While we observed no statistically significant differences in Cx32 expression between PD and control cases within the putamen (Fig. 7g), the SNpc, a region highly vulnerable to PD pathogenesis, showed a marked decrease in Cx32 expression (Fig. 7h). Interestingly and consistent with the decrease in Cx32 within the substantia nigra, we observed no significant differences in the levels of α-syn from control and PD cases from the same region (Suppl. Figure S9c, Online Resource 12). We then assessed the levels of Cx32 in an area outside the nigrostriatal pathway, the Brodmann area 9 (BR9) of the dorsal lateral pre-frontal cortex, a region relatively unaffected by α-syn pathology in PD during the early stages of the disease process. In contrast to the substantia nigra, we observed no significant differences in Cx32 protein levels between controls (*n* = 15) and PD cases (*n* = 15) (Fig. 7i, j), suggesting that the reduction of Cx32 levels within the nigral region may serve as a protective mechanism to reduce α-syn uptake and accumulation. To further validate the interaction between Cx32 and α-syn in human PD brains, we next immunoprecipitated α-syn from the putamen, the region containing higher levels of Cx32 relative to the SNpc. Cx32 co-immunoprecipitated with α-syn in two out of four PD cases but this interaction was not observed in any of the four age-matched control cases tested (Fig. 7k, Suppl. Figure S9d, Online Resource 12). We next analyzed Cx32 levels from the putamen region of control and pathologically diagnosed MSA cases (Suppl. Table S3, Online Resource 3), a region highly vulnerable to α-syn accumulation in oligodendrocytes of MSA patients [54, 64]. Similar to the substantia nigra in PD, we identified a significant decrease in Cx32 levels in MSA cases compared to age-matched controls (Fig. 7l), further validating a potential relationship between human α-syn and Cx32 in the pathophysiology of PD and MSA.

## Discussion

In this study, we demonstrate that Cx32 plays a critical role in facilitating the preferential uptake of oα-syn via direct protein–protein interaction in both neurons and oligodendrocytes, the primary cell types highly vulnerable to α-syn accumulation in PD and MSA, respectively. Our findings contribute to the current hypothesis on the progression and spreading of neurodegenerative disorders, which highlights the importance of cellular connectivity in the uptake and cell-to-cell transfer of misfolded proteins. It is well-established that cellular communication is enhanced by Cx expression, and while novel functional roles outside of neuronal communication have been identified for other Cxs [19], the functional roles of Cxs in the progression of PD and MSA have remained relatively unexplored. To our knowledge, the current study demonstrates for the first time that Cx32 plays an essential role in the uptake and propagation of oα-syn assemblies, and to a lesser extent monomers and fibrillar assemblies. It is important to note that the limited uptake of α-syn fibrils mediated by Cx32 observed in this study may in part be due to the lack of fibril sonication. Sonication not only reduces the overall fibrillar length, but also generates oligomeric α-syn assemblies, and induces unwanted post-translational modifications within the molecule [1, 38]. Therefore, to maintain fibril integrity and avoid a heterogeneous mixture of oligomeric and fibrillar assemblies in our fibrillar preparations, we omitted the sonication step. Given this notion, we are aware that further studies are needed to assess whether fibril sonication might increase the uptake of fragmented fibrillar assemblies by Cx32 [1, 2]. Importantly, while the previous studies have emphasized Cx32 expression within cells of the oligodendrocyte lineage, more recent reports suggest Cx32 expression within multiple neuronal cell types, including those from cortical and dopaminergic regions [17, 49, 50]. Furthermore, single cell RT-PCR analyses validated Cx32 expression within neurons from the locus coeruleus, cortex, hippocampus and substantia nigra, a brain region highly vulnerable to PD pathogenesis [69]. Moreover, Gong and colleagues validated the presence of Cx32 expression within primary cortical neurons using siRNA technology [17]. Taken together, these results validate the expression profile of Cx32 within multiple neuronal cell types.

To further expand these findings, we demonstrate herein that Cx32 is differentially regulated in neurons and oligodendrocytes both in vitro and in animal models of PD (L61, A30P) and MSA (MBP29). Whereas upregulation of Cx32 was observed in human neuronal SH-SY5Y cells exposed to oα-syn assemblies or by overexpressing the α-syn gene (*SNCA*), only exposure to oα-syn in naïve oligodendrocytes increased Cx32 expression (albeit to a lesser extent than in neurons). Conversely, expression of α-syn-GFP coupled with oα-syn treatment in OPCs led to a significant decrease in Cx32 expression compared to that of wild-type OPC-treated cells. We hypothesize that this may be due to differential regulation of Cx32 within OPCs compared to their neuronal counterpart. Indeed, OPCs overexpressing Cx32-mCherry showed a significant downregulation of Cx32 following oα-syn treatment, which we observed to be upregulated in neuronal cells and wild-type OPCs exposed to the same conditions. The downregulation of Cx32 in oligodendrocytes may also be explained by its direct association with oligodendrocyte myelination, as we have previously demonstrated that α-syn expression within oligodendrocytes leads to oligodendrocyte maturation deficits [12]. While the expression pattern of Cx32 as it relates to myelination remains to be determined, gene expression analysis has identified Cx32 expression primarily within myelinating oligodendrocytes [74], suggesting a direct association between Cx32 expression and oligodendrocyte myelination. While the factors that regulate Cx32 expression in neurons and oligodendrocytes remain elusive, we have demonstrated that the increased expression of Cx32 in neurons and oligodendrocytes leads to a selective increase in oα-syn uptake compared to that of α-syn monomers or fibrillar assemblies.

Notably, although the levels of oα-syn uptake in rat OLN-93 oligodendrocytes were less than those observed in SH-SY5Y cells or human oligodendrocytes, the inability of Cx32 to properly localize to the cellular membrane in OLN-93 cells may, in part, explain this result. For instance, in liver hepatocytes, the cell type with the highest expression of Cx32 outside the central nervous system, Cx32 is localized to the cell soma in immature cells, before it fully translocates to the cellular membrane following hepatocyte maturation [39]. Consistent with this observation, Buckinx and colleagues have reported that OLN-93 oligodendrocytes lack the ability to fully differentiate in vitro and, as such, fail to express the protein markers associated with mature oligodendrocytes [6]. These findings suggest that OLN-93 oligodendrocytes in our culture conditions may not reach full maturation and, as such, may incorrectly compartmentalize Cx32, preventing the interaction between Cx32 and oα-syn necessary to facilitate cellular uptake. This hypothesis is consistent with our experimental results using anisomycin, the p38 MAPK activator known to promote Cx32 degradation [39], leading to a significant decrease in oα-syn uptake. These results demonstrate that proper localization of Cx32 is necessary to interact with oα-syn prior to cellular uptake.

To demonstrate a direct protein–protein interaction between Cx32 and oα-syn, and eliminate potential antibody cross-reactivity, we took advantage of our tagged Cx32-mCherry system and developed a co-culture model that allows for direct quantification of α-syn cell-to-cell transfer, a model that is suitable for high content analysis [43]. Using this system, we successfully quantified higher levels of α-syn-GFP puncta in cells expressing Cx32-mCherry relative to the respective controls. Using confocal image analysis, we validated the colocalization between α-syn-GFP oligomers and Cx32-mCherry plaques, indicating that a direct interaction between Cx32 and α-syn is necessary for binding and internalization. Moreover, we demonstrated that α-syn cell-to-cell transfer in human SH-SY5Y cells is enhanced by direct cellular connectivity rather than its differentiation state. Nonetheless, the observed increase in oα-syn transfer in differentiated conditions is likely an effect of enhanced cellular connectivity and not due to an elevated α-syn load, as no statistically significant differences were observed in α-syn transfer in the α-syn-GFP/mCherry co-culture model in proliferating vs differentiating conditions. Recently, the viral HIV protein dickkopf-1 (26 kDa) was shown to promote uptake and downstream toxicity by inducing the opening of Cx43 hemichannels [32]. A pore-forming activity for α-syn has been reported [65], suggesting a potential mechanism similar to that seen with dickkopf-1 for oα-syn uptake and transfer via Cx32. Nonetheless, it is clear that a direct interaction between oα-syn and Cx32 enhances oα-syn uptake in neurons and oligodendrocytes. Indeed, expanding on previous in vitro results [60], we further demonstrate that IP of oα-syn in differentiated SH-SY5Y cells pulls down Cx32 and that the levels of α-syn correlate with Cx32 upregulation, pinpointing a direct relationship between Cx32 and α-syn. These results suggest that oα-syn uptake is mediated, at least in part, via a protein–protein interaction, and not due to non-specific membrane binding or Cx32-dependent channel diffusion, as we have shown this mechanism to be energy-dependent. Our work highlights the role of Cx proteins and neuronal connectivity during misfolded protein uptake and transfer, which we believe may extend to other neurodegenerative disorders beyond PD and related synucleinopathies which includes AD as shown by the uptake of oAβ assemblies mediated by Cx32.

In an attempt to block cellular α-syn uptake, we used well-known Cx32 peptide mimetics that bind and inhibit Cx32 hemichannel activity (Gap3211 and Gap2409). While we observed a partial inhibition of oα-syn uptake in neurons and oligodendrocytes, this effect did not translate to fibrillar assemblies. In contrast to these results, the use of gap junction inhibitors (CBX, MQ and 2-APB) successfully blocked soluble and insoluble (fibrillar) α-syn uptake in both neurons and oligodendrocytes. The lack of fibrillar uptake inhibition by peptide mimetics may in part be explained by the high affinity of fibrillar α-syn assemblies to cellular membranes [18], the selective binding of Cx32 to oα-syn, the limited efficacy of the Cx32 peptide sequences used (which may not target the region interest) and/or the half-life of the peptides within the cells. However, other mechanisms cannot be ruled out including an unidentified Cx that may be involved in the preferential uptake of fibrillar α-syn assemblies. The recent identification of LAG-3, a specific receptor for the uptake and transmission of α-syn fibrils, suggest different mechanisms of uptake between oligomeric and fibrillar assemblies [29]. However, oligodendrocytes lack LAG-3 expression [29] and, therefore, the involvement of other Cx proteins in the selective uptake of different α-syn assemblies remains plausible. Collectively, these results indicate that pharmacological strategies may be more effective than peptide mimetics at blocking the uptake of oligomeric and fibrillar assemblies in neurons and oligodendrocytes via a Cx32-dependent mechanism.

The accumulation of α-syn pathology and subsequent propagation in PD and MSA, as well as corresponding animal models of these disorders, display a clear age-dependent association. Thus, the observed increase in Cx32 protein levels between adult and aged Tg PD models (L61, A30P), as well as those modeling MSA (MBP29), are consistent with the increase in α-syn pathology within vulnerable brain regions of these mice over time [22, 46]. However, further studies are needed to assess whether the familial A30P mutation, given its lower affinity for cellular membranes [66], exacerbates Cx32 expression compared to its wild-type counterpart. Furthermore, the binding affinities of A30P and other familial α-syn mutations to Cx32 and/or their ability to promote Cx32 upregulation compared to wild-type α-syn remains to be determined. Nonetheless, all PD and MSA animal models assessed in this study displayed an age-dependent upregulation of Cx32 which is likely due to the increased α-syn accumulation in the brain parenchyma, directly implicating Cx32 in the pathophysiology of PD and MSA. In human brains, however, a significant decrease in Cx32 levels within the substantia nigra and the putamen region of PD and MSA cases, respectively, was clearly observed. Although the reasons for the decrease in Cx32 levels within the primary regions affected by PD (substantia nigra) or MSA (putamen) remain relatively unknown, the discrepancy between the cellular and animal models compared to human PD may be explained by multiple factors, including the degree of α-syn pathology and/or the region associated with α-syn vulnerability, coupled to cell loss within affected brain regions [54, 64]. Indeed, we analyzed a region relatively unaffected by α-syn accumulation during the early stages of the disease process (the BR9 cortical region) in both control and PD cases and observed no significant changes in Cx32 expression. Therefore, it is plausible that the reduction in Cx32 levels may be a cellular attempt to reduce α-syn accumulation in regions with high α-syn burden as demonstrated by our human oligodendrocyte model system. Thus, the regions that lose Cx32 in response to PD or MSA (e.g., substantia nigra or putamen, respectively) may be more vulnerable to cell death due to the accumulation of α-syn pathology. Interestingly, increased Cx32 expression has been demonstrated to promote cellular apoptosis [68], suggesting that, in addition to increasing cellular connectivity and modulating α-syn uptake, Cx32 may have other non-canonical functions that remain to be fully elucidated. Moreover, given that we identified a direct interaction between Cx32 and α-syn in PD, but not in control cases, suggests the possibility that Cx32 may associate within different α-syn assemblies (SDS-insoluble) during the aggregation process in PD. Alternatively, it is possible that the expression profile of non-neuronal cell types (which differentially regulate Cx32 expression in pathological state) may mask the true expression of Cx32 in neurons in PD and MSA. Therefore, we hypothesize that Cx32 upregulation may first occur during the early stages of the disease as shown for other Cx in different neurodegenerative diseases [49], followed by a downregulation in late stages as a protective mechanism to reduce the uptake and accumulation of α-syn pathology; however, additional studies are needed to validate this hypothesis.

To date, multiple model systems have been developed to assess the uptake and transmission of different α-syn assemblies in vitro and in vivo [41, 42, 45]. Recently, Fortuna and colleagues demonstrated that intra-cerebroventricular injection of oα-syn assemblies in non-Tg wild-type mice is able to induce the PD-associated motor and non-motor symptoms that were previously demonstrated following striatal injection of fibrillar α-syn assemblies [14, 28, 52, 53]. While the changes in the Cx expression in these transmission models of α-syn assemblies have yet to be investigated, we hypothesize that Cx32 plays a major role in this process and may contribute and/or dictate the direction and/or cell-type vulnerability of α-syn spreading from one region to another. Although the role of Cx32 upregulation as a result of α-syn accumulation remains elusive, we further hypothesize that Cx32 in neurons plays a role in the bystander effect associated with the spread of toxic signals, as reported for other Cxs in critical brain regions in other neurodegenerative disorders [49]. For instance, Cx43 upregulation was demonstrated within the caudate nucleus in Huntington’s disease (HD) and within the cortex in AD [30, 72]. Moreover, in cultured astrocytes, Cx43 appears to be upregulated following oAβ treatment [33]. However, a similar role for Cx43 in neurons was not identified but, a different, unidentified Cx has been hypothesized to play a similar role [33]. In global ischemia, vulnerable neurons of the CA1 region were found to increase Cx32 and Cx36 expression, whereas no changes in the CA3 region were observed, suggesting vulnerable regional specificity associated with Cx upregulation in the disease state [31]. Taken together, our results show a novel mechanism selective for the uptake and propagation of oα-syn assemblies mediated by Cx32. Moreover, we demonstrate that Cx32 is a suitable therapeutic candidate for pharmacological intervention to impede the uptake and propagation of α-syn assemblies in PD and MSA.

## Electronic supplementary material

Below is the link to the electronic supplementary material.

Supplementary material 1 (PDF 68 kb)

Supplementary material 2 (PDF 184 kb)

Supplementary material 3 (PDF 213 kb)

Supplementary material 4 (TIFF 16680 kb) **Suppl. Figure S1 (Online Resource 4). Cx32 localizes to the cellular membrane and facilitates the uptake of oα-syn assemblies**. **a** TEM images of α-syn oligomers and **b** fibrils using the same magnification. **c** TEM quantification of α-syn fibrils with an average length of 133.7 nm ± 3.53 (n = 630 filaments counted from 10 different images). **d** Densitometry analysis of mCherry and GFP proteins following low (15 µg) or high (50 µg) expression of Cx32-mCherry and Cx43-GFP in HEK-293 cells. **e**Confocal image analysis of HEK-293 cells expressing either Cx32-mCherry or **f** Cx43-GFP showing the localization at the cellular membrane (arrowheads), scale bars 10 µm. **g** Densitometry analysis of HEK-293 cells expressing high levels (50 µg) of Cx32 or Cx43 incubated with monomeric, oligomeric or fibrillar α-syn assemblies for 24 h (n = 3, two-way ANOVA followed by Tukey’s post hoc test for multiple comparisons, *F*_(8, 16)_ = 259.3, **p < 0.01, ***p < 0.001, ****p < 0.0001). **h** Densitometry analysis of monomeric α-syn uptake in WT differentiated SH-SY5Y cells or SH-SY5Y cells expressing Cx26, Cx32, Cx43 and Cx32-KO, (n = 5, one-way ANOVA followed by Tukey’s post hoc test for multiple comparisons, n.s; no significance, *F*_(4, 15)_ = 119.6, ****p < 0.0001). **i** Densitometry analysis of oα-syn uptake in differentiated SH-SY5Y Cx32-mCherry and Cx32-KO cells (n = 4, unpaired, two-tailed *t* test, *t*_5_ = 16.67, ****P < 0.0001).

Supplementary material 5 (TIFF 16683 kb) **Suppl. Figure S2 (Online Resource 5). Cellular connectivity increases α-syn cell-to-cell transfer**. **a** Representative confocal micrographs of differentiated SH-SY5Y donor and recipient co-cultures expressing α-syn-GFP (donor cells, green) and mCherry (acceptor cells, red), and immunolabeled with β3-tubulin antibodies (gray) and Hoechst (blue). **b** Differentiated SH-SY5Y co-cultures expressing α-syn-GFP (donor cells, green) and Cx32-mCherry (acceptor cells, red), and immunolabeled with β3-tubulin antibodies (gray) and Hoechst (blue). Scale bars represent 100 µm and 50 µm for low and high magnification, respectively. **c** Flow cytometry of undifferentiated SH-SY5Y cells, depicting α-syn-GFP transfer after 5 days (n = 4, independent experiments, one-way ANOVA, *F*_(3, 12)_ = 910, n.s. = non-significant, ****p < 0.0001. **d** Confocal image analysis of FACS-sorted recipient cells expressing either mCherry or Cx32-mCherry, and containing α-syn-GFP oligomers, analyzed with HuygensPro to indicate a clear colocalization between Cx32 and α-syn-GFP, Pearson correlation coefficient of P = 0.032 and 0.159 for α-syn-GFP and mCherry compared to P = 0.668 and 0.696 for Cx32-mCherry and α-syn-GFP.

Supplementary material 6 (TIFF 16647 kb) **Suppl. Figure S3 (Online Resource 6). Cx32-mCherry colocalizes with oα-syn in human cells of the oligodendrocyte lineage.a** Confocal image analysis of OPCs undergoing differentiation into mature oligodendrocytes, expressing the oligodendrocyte-specific marker CNPase (green) but lack MBP expression at 15 days of differentiation followed by Hoechst staining (blue). **b** Confocal image analysis of OPCs on day 30 of differentiation into mature oligodendrocytes, expressing the oligodendrocyte-specific marker MBP (green) and stained with Hoechst (blue). **c** Confocal image analysis of human OPCs (CNPase + , gray) overexpressing Cx32-mCherry (red) shows a high degree of colocalization as noted by the merging of Cx32-mCherry (red) and oα-syn-ATTO-488 (green); stained with Hoechst (blue). Scale bars represent 50 µm. **d** IP of oα-syn followed by Western blotting with Cx32 antibody shows an interaction between Cx32 (arrow) and oα-syn in human OPCs.

Supplementary material 7 (TIFF 5126 kb) **Suppl. Figure S4 (Online Resource 7). Cx32 peptide mimetics block oα-syn uptake but not fibrillar assemblies.a** Fluorescence intensity measurements of α-syn-ATTO-550 uptake in differentiated SH-SY5Y cells exposed to oα-syn or fibrillar assemblies in the presence of Gap2409 peptide mimetics, (n = 12, one-way ANOVA followed by Tukey’s post hoc test for multiple comparisons, n.s; no significance*, F*_(5, 68)_ = 13.33, ****p < 0.0001). **b** Fluorescence intensity measurements of α-syn-ATTO-550 uptake in differentiated OLN-93 oligodendrocyte cells exposed to oα-syn or fibrillar assemblies in the presence of Gap2409 peptide mimetics (n = 12, one-way ANOVA followed by Tukey’s post hoc test for multiple comparisons, n.s; no significance*, F*_(5, 66)_ = 9.392, **p < 0.01, ****p < 0.0001). **c** Representative confocal micrographs of differentiated SH-SY5Y cells exposed to oligomeric assemblies. (d) Representative confocal micrographs of differentiated OLN-93 oligodendrocyte cells exposed to oligomeric assemblies. **e** Representative confocal micrographs of differentiated SH-SY5Y cells exposed to fibrillar assemblies **f** Representative confocal micrographs of differentiated OLN-93 oligodendrocyte cells exposed to fibrillar assemblies. Cells were immunolabeled with the neuron-specific β3-tubulin (green), α-syn-ATTO-550 (red), Hoechst (blue) and visualized using confocal microscopy. Scale bars represent 100 µm.

Supplementary material 8 (TIFF 5920 kb) **Suppl. Figure S5 (Online Resource 8). Scrambled Cx32 or Cx43 peptide mimetics have no effect on oligomeric or fibrillar α-syn uptakea** Fluorescence intensity measurements of α-syn-ATTO-550 uptake in differentiated human SH-SY5Y cells exposed to oα-syn or fibrillar assemblies in the presence of functional and scrambled (SC) Gap3211 peptide mimetics, (n = 12, one-way ANOVA followed by Tukey’s post hoc test for multiple comparisons, n.s; no significance*, F*_(7, 80)_ = 7.044, ****p < 0.0001). **b** Fluorescence intensity measurements of α-syn uptake in differentiated SH-SY5Y cells exposed to oα-syn or fibrillar assemblies in the presence of functional Cx43 (Gap2605) peptide mimetics, (n = 12, one-way ANOVA followed by Tukey’s post hoc test for multiple comparisons, n.s; no significance; *F*_(5, 66)_ = 0.6833).

Supplementary material 9 (TIFF 3158 kb) **Suppl. Figure S6 (Online Resource 9). The pan-Cx specific gap junction inhibitor carbenoxolone (CBX) blocks the uptake of oligomeric and fibrillar α-syn assembliesa** Fluorescence intensity measurements of α-syn-ATTO-550 uptake in differentiated human SH-SY5Y cells exposed to oα-syn or fibrillar assemblies in the presence of increasing concentrations of the pan-specific gap junction inhibitor CBX (n = 9, of 3 independent experiments one-way ANOVA followed by Tukey’s post hoc test for multiple comparisons, n.s; no significance, *F*_(5, 45)_ = 76.6, *p < 0.05, **p < 0.01, ****p < 0.0001). **b** Representative confocal micrographs of differentiated SH-SY5Y cells co-labeled with β3-tubulin (green), α-syn (ATTO-550) and Hoechst (blue) visualized using confocal microscopy. **c** Fluorescence intensity measurements of oα-syn and fibrillar uptake in differentiated OLN-93 oligodendrocytes in the presence of CBX (n = 16 in 4 independent experiments, one-way ANOVA followed by Tukey’s post hoc test for multiple comparisons, n.s; no significance, *F*_(5, 90)_ = 14.31, ***p < 0.001, ****p < 0.0001). **d** Representative confocal micrographs of differentiated OLN-93 oligodendrocytes co-labeled with α-syn (ATTO-550) and Hoechst (blue) visualized using confocal microscopy. Scale bars represent 100 µm.

Supplementary material 10 (TIFF 7483 kb) **Suppl. Figure S7 (Online Resource 10). The selective gap junction inhibitor mefloquine (MQ) blocks the uptake of oligomeric and fibrillar α-syn assemblies.a** Fluorescence intensity measurements of oα-syn and fibrillar uptake in differentiated SH-SY5Y cells in the presence of the selective gap junction inhibitor MQ, (n = 12, one-way ANOVA followed by Tukey’s post hoc test for multiple comparisons, *F*_(5, 66)_ = 29.18, *p < 0.05, **p < 0.01, ***p < 0.001, ****p < 0.0001). **b** Representative micrographs of differentiated human SH-SY5Y cells co-labeled with β3-tubulin (green), α-syn-ATTO-550 (red) and Hoechst (blue) visualized under confocal microscopy. **c** Fluorescence intensity measurements of oα-syn and fibrillar uptake in primary cortical neurons (DIV 10) in the presence of MQ, (n = 12, one-way ANOVA followed by Tukey’s post hoc test for multiple comparisons, n.s; no significance*, F*_(5, 66)_ = 12.46, *p < 0.05, ***p < 0.001, ****p < 0.0001). **d** Representative micrographs of primary cortical neurons co-labeled with β3-tubulin (green), α-syn-ATTO-550 (red) and Hoechst (blue). **e** Fluorescence intensity measurements of oα-syn and fibrillar uptake in differentiated OLN-93 oligodendrocytes in the presence of MQ, (n = 12, one-way ANOVA followed by Tukey’s post hoc test for multiple comparisons, *F*_(5, 42)_ = 35.33, **p < 0.01, ***p < 0.001, ****p < 0.0001). Representative confocal micrographs of differentiated OLN-93 oligodendrocytes co-labeled with α-syn-ATTO-550 (red) and Hoechst (blue) visualized using confocal microscopy. Scale bars represent 100 µm.

Supplementary material 11 (TIFF 938 kb) **Suppl. Figure S8 (Online Resource 11)**. **Cx32 upregulation correlates with human α-syn expression.a** Western blot of differentiated SH-SY5Y cells expressing α-syn-GFP exposed to oα-syn assemblies shows the uptake of oα-syn assemblies and the lack of α-syn-GFP degradation in untreated α-syn-GFP cells. **b** Confocal micrographs of low- and high-expression α-syn-GFP cells sorted by FACS, and co-labeled with α-syn antibody (red) and Hoechst (blue). Scale bars represent 20 µm. **c** Western blot analysis of Cx32 protein levels in differentiated SH-SY5Y cells expressing low (n = 11) or high α-syn-GFP (n = 4, unpaired, two-tailed *t* test*, t*_*1*3_ = 4.862, ***p < 0.001). **d** Cx32 mRNA fold change of differentiated SH-SY5Y cells expressing low (n = 7) and high levels (n = 8) of α-syn-GFP and wild-type non-transfected cells as controls (one-way ANOVA followed by Tukey’s post hoc test for multiple comparisons, *F*_(2, 16)_ = 11.81 **p < 0.007). **e** Correlation analysis between the levels of α-syn and Cx32 protein expression in differentiated SH-SY5Y cells (R^2^ = 0.9017, ***p < 0.001). **f** Western blot analysis of Cx32 proteins in non-Tg and Tg cohorts expressing human wild-type α-syn (L61, n = 5, Mann–Whitney test *U *= 5, n_1_ = n_2_ = 5 *p < 0.05). **g** Western blot analysis of Cx32 in non-Tg and Tg cohorts expressing human α-syn proteins harboring the A30P mutation (A30P) using C-terminal region antibodies to Cx32 (Mann–Whitney test *U *= 3, n_1_ = n_2_ = 6 *p < 0.05). **h** Cx32 mRNA fold change of non-Tg and Tg-L61 α-syn (n = 5) or mutant-A30P α-syn and age-matched control cohorts (n = 6, one-way ANOVA followed by Tukey’s post hoc test for multiple comparisons, n.s; no significance*, F*_(3, 18)_ = 0.4887).

Supplementary material 12 (TIFF 16224 kb) **Suppl. Figure S9 (Online Resource 12)**. **Cx32 interacts with α-syn in human PD brains.a** Confocal image analysis of tissue sections from aged A30P mice immunolabeled with the oligodendrocyte-specific antibody CNPase (green), and α-syn (red; I–IV), or CNPase (red) and α-syn phosphorylated at serine-129 (green; V–VIII), scale bar 10 µm. **b**. Densitometric analysis of Cx32 protein levels in young ~ 1-month (3 weeks) and adult Tg (3 months) MBP29 cohorts from the cortex and corpus callosum (n = 5, one-way ANOVA followed by Tukey’s post hoc test for multiple comparisons, *F*_(3, 16)_ = 16.13, **p < 0.01, ***p < 0.001). **c** Densitometry analysis of human α-syn within the nigra of control and PD cases (unpaired two-tailed *t* test, n.s. = no significance). **d** IP of α-syn from human PD cases and age-matched controls followed by Western blot analysis. Note that Cx32 is identified in PD cases (total: 2 out of 4) but not in controls (total: 0 out 4).
