# Supplementary material for: Binding of α-synuclein oligomers to Cx32 facilitates protein uptake and transfer in neurons and oligodendrocytes
Source: Acta Neuropathol. 2019 Apr 11;138(1):23–47. doi: 10.1007/s00401-019-02007-x (PMC6570706; doi:10.1007/s00401-019-02007-x)
Supplement: Supplementary file 1 — Supplementary material 1 (PDF 68 kb) [file 401_2019_2007_MOESM1_ESM.pdf]

Table S3. Primary antibodies, primers and gRNA sequences used in this study

Antibodies/Genes	Company/ ID	Dilution
CNPase antibody<br>(Mouse, Rabbit)	Invitrogen: PA5-29345<br>Sigma: SAB4200693	IHC, 1/250<br>IHC, 1/250
Cx32 antibody (Mouse)	Millipore: MAB3069	WB, 1/1000<br>IHC, 1/250
Cx32 antibody (Rabbit)	Thermo Scientific: 71-0600	IHC, 1/250
Cx32 antibody: (Mouse)	Abcam: 66613	WB, 1/1000<br>IHC, 1/250
α-syn antibody: (Rabbit)	Invitrogen: 701085	WB, 1/20,000<br>IHC, 1/5,000
pS129 α-syn antibody<br>(Mouse)	Wako: 015-25191	IHC, 1/1,000
GAPDH antibody:<br>(Mouse)	Novus Biologicals: 2D4A7	WB, 1/10,000
ß3-tubulin antibody:<br>(Mouse, Rabbit)	Sigma Aldrich: Tuj1<br>Biolegend: (Poly18020)	IHC, 1/1,000
ß-actin antibody: (Mouse)	Sigma Aldrich: AC-15	WB, 1/10,000
mCherry antibody:<br>(Rabbit)	Invitrogen: PA5-34974	WB, 1/1000<br>IHC, 1/250
Aß antibody: (Mouse)	Biolegend: 6E10	WB, 1/1000<br>IHC, 1/250
MBP: (Mouse)	Santa Cruz Biotechnology: 271524	IHC, 1/250
GJA1	Hs00748445	
GJA1	Mm00439105	
GAPDH	Hs03929097	
GAPDH	Mm99999915	
GJB1	Hs00939759	
GJB1	Ms00439759	
SNCA	Hs01103383	
CRISPR gRNA sequence	GTGGACCTATGTCATCAGCG	Pam: TGG
