# Supplementary material for: Binding of α-synuclein oligomers to Cx32 facilitates protein uptake and transfer in neurons and oligodendrocytes
Source: Acta Neuropathol. 2019 Apr 11;138(1):23–47. doi: 10.1007/s00401-019-02007-x (PMC6570706; doi:10.1007/s00401-019-02007-x)
Supplement: Supplementary file 2 — Supplementary material 2 (PDF 184 kb) [file 401_2019_2007_MOESM2_ESM.pdf]

Table S1. Transgenic animal models used in this study

Line	Age (months)	Sex
L61	6M	M
L61	6 M	M
L61	6M	M
L61	6 M	M
L61	6M	M
A30P	18	M
A30P	18	M
A30P	17.5	M
A30P	18	F
A30P	18	F
A30P	18	F
MBP-29	1 M	M
MBP-29	1 M	M
MBP-29	1 M	M
MBP-29	1 M	M
MBP-29	1 M	M
MBP-29	3 M	M
MBP-29	3 M	M
MBP-29	3 M	M
MBP-29	3 M	M
MBP-29	3 M	M
