# Supplementary material for: Binding of α-synuclein oligomers to Cx32 facilitates protein uptake and transfer in neurons and oligodendrocytes
Source: Acta Neuropathol. 2019 Apr 11;138(1):23–47. doi: 10.1007/s00401-019-02007-x (PMC6570706; doi:10.1007/s00401-019-02007-x)
Supplement: Supplementary file 3 — Supplementary material 3 (PDF 213 kb) [file 401_2019_2007_MOESM3_ESM.pdf]

Table S2. Demographics of human brain samples of PD, MSA and control cases used for this study

Diagnosis	Age of Death	Gender	PMD	Age at diagnosis	Region analyzed	ID #
Control	80	M	25	NA	Putamen/nigra	56490
Control	73	M	14	NA	Putamen/nigra	18589
Control	70	F	32	NA	Putamen/nigra	16391
Control	77	M	22	NA	Putamen/nigra	74890
PD, mild AD, Congo Angiopathy	80	F	14	69	Putamen/nigra	12595
PD, LBD	70	M	7.5	PD-64, AD-69	Putamen/nigra	6013
PD	78	M	16	66	Putamen/nigra	7815
PD	75	M	28	63	Putamen/nigra	64891
Control	82	F	43	N/A	Cortex	A011/06
Control	87	F	21.5	N/A	Cortex	A047/02
Control	81	M	42	N/A	Cortex	A048/09
Control	79	M	34	N/A	Cortex	A049/03
Control	90	F	74	N/A	Cortex	A063/10
Control	85	M	48	N/A	Cortex	A133/95
Control	86	M	6	N/A	Cortex	A134/00
Control	89	F	65	N/A	Cortex	A136/10
Control	71	M	5	N/A	Cortex	A153/01
Control	68	F	9	N/A	Cortex	A170/00
Control	80	M	48	N/A	Cortex	A185/04
Control	80	M	11	N/A	Cortex	A223/96
Control	79	F	38	N/A	Cortex	A239/95
Control	77	M	29	N/A	Cortex	A283/96
Control	66	M	52	N/A	Cortex	A308/09
Control	80	M	35	N/A	Cortex	A316/95
Control	77	M	96	N/A	Cortex	A320/94
Control	96	F	72	N/A	Cortex	A33/96
Control	85	M	16	N/A	Cortex	A346/95
Control	80	F	22	N/A	Cortex	A359/08
Control	85	M	42	N/A	Cortex	A401/97
Control	65	M	29	N/A	Cortex	A61/96
PD	62	F	41	N/A	Cortex	BBN_15746
PD	89	F	54	N/A	Cortex	BBN_15613
PD	69	F	34	N/A	Cortex	BBN_16484
PD	85	F	9	N/A	Cortex	BBN_15696
PD	73	M	40	N/A	Cortex	BBN_16602

PD	76	M	66	N/A	Cortex	BBN_16605
PD	72	M	29	N/A	Cortex	BBN_16638
PD	80	F	72	N/A	Cortex	BBN_16644
PD	79	M	89	N/A	Cortex	BBN_16657
PD	59	M	23	N/A	Cortex	BBN_16661
PD	74	M	10	N/A	Cortex	BBN_16683
PD	84	M	34	N/A	Cortex	BBN_15702
PD	81	F	38	N/A	Cortex	BBN_16754
PD	73	F	82	N/A	Cortex	BBN_16797
PD	76	F	47	N/A	Cortex	BBN_16854
PD	70	M	49	N/A	Cortex	BBN_16856
PD	75	M	40	N/A	Cortex	BBN_16917
**Diagnosis**	**Age of death**	**Sex**	**PMD**	**Age at diagnosis**	**Region analyzed**	**ID #**
Control	55	F	5.35	N/A	Putamen	N/A
Control	60	F	7.3	N/A	Putamen	N/A
Control	64	F	5.4	N/A	Putamen	N/A
Control	51	M	7.45	N/A	Putamen	N/A
Control	55	M	7.15	N/A	Putamen	N/A
MSA	66	F	8.05	N/A	Putamen	N/A
MSA	67	F	7.15	N/A	Putamen	N/A
MSA	59	F	6.4	N/A	Putamen	N/A
MSA	55	M	8.4	N/A	Putamen	N/A
MSA	67	M	6.1	N/A	Putamen	N/A
