# Supplementary figures and images for: Binding of α-synuclein oligomers to Cx32 facilitates protein uptake and transfer in neurons and oligodendrocytes
Source: Acta Neuropathol. 2019 Apr 11;138(1):23–47. doi: 10.1007/s00401-019-02007-x (PMC6570706; doi:10.1007/s00401-019-02007-x)

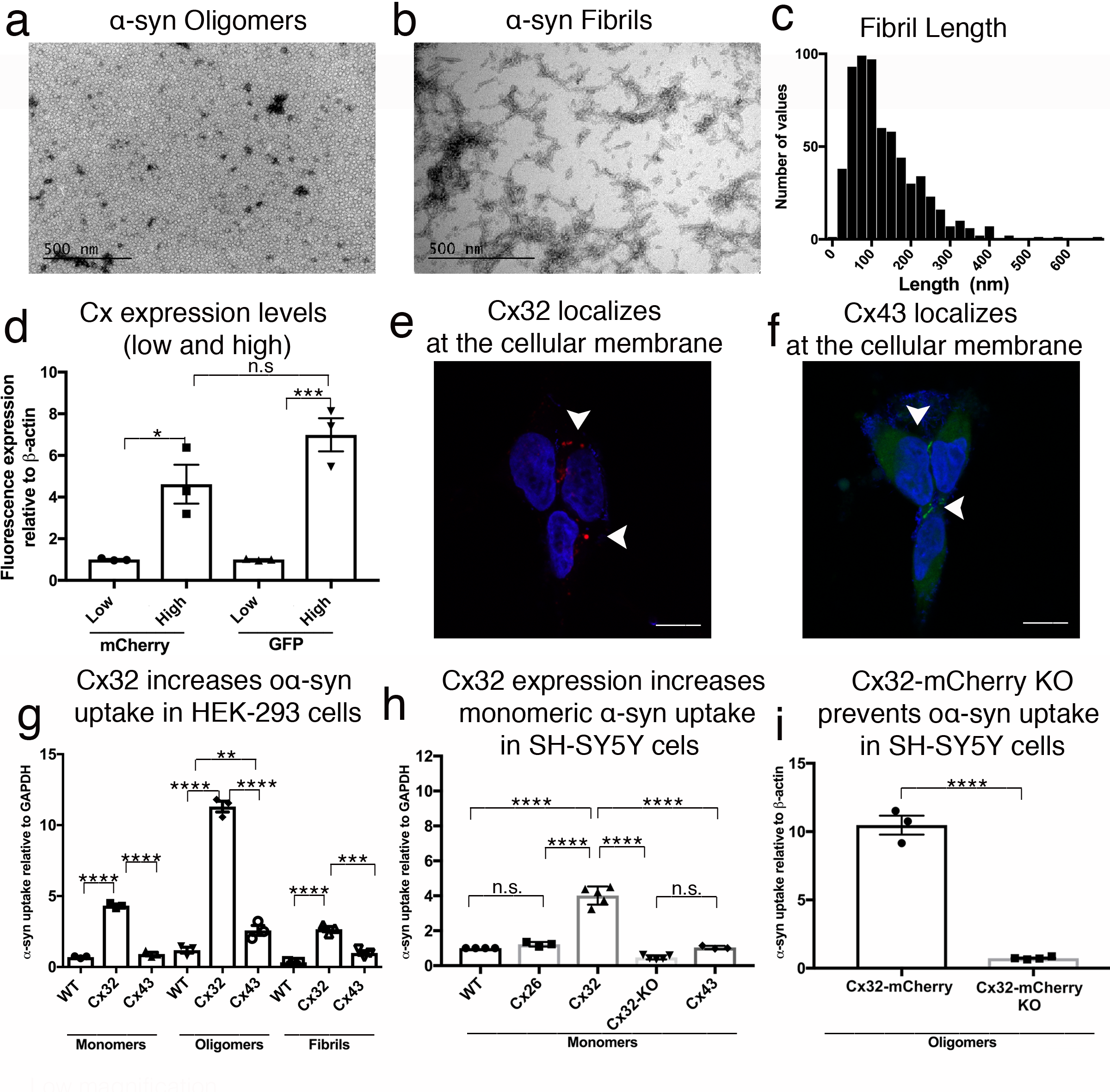

Supplement: Supplementary file 4 — Supplementary material 4 (TIFF 16680 kb) Suppl. Figure S1 (Online Resource 4). Cx32 localizes to the cellular membrane and facilitates the uptake of oα-syn assemblies. a TEM images of α-syn oligomers and b fibrils using the same magnification. c TEM quantification of α-syn fibrils with an average length of 133.7 nm ± 3.53 (n = 630 filaments counted from 10 different images). d Densitometry analysis of mCherry and GFP proteins following low (15 µg) or high (50 µg) expression of Cx32-mCherry and Cx43-GFP in HEK-293 cells. eConfocal image analysis of HEK-293 cells expressing either Cx32-mCherry or f Cx43-GFP showing the localization at the cellular membrane (arrowheads), scale bars 10 µm. g Densitometry analysis of HEK-293 cells expressing high levels (50 µg) of Cx32 or Cx43 incubated with monomeric, oligomeric or fibrillar α-syn assemblies for 24 h (n = 3, two-way ANOVA followed by Tukey’s post hoc test for multiple comparisons, F(8, 16) = 259.3, **p < 0.01, ***p < 0.001, ****p < 0.0001). h Densitometry analysis of monomeric α-syn uptake in WT differentiated SH-SY5Y cells or SH-SY5Y cells expressing Cx26, Cx32, Cx43 and Cx32-KO, (n = 5, one-way ANOVA followed by Tukey’s post hoc test for multiple comparisons, n.s; no significance, F(4, 15) = 119.6, ****p < 0.0001). i Densitometry analysis of oα-syn uptake in differentiated SH-SY5Y Cx32-mCherry and Cx32-KO cells (n = 4, unpaired, two-tailed t test, t5 = 16.67, ****P < 0.0001). [file 401_2019_2007_MOESM4_ESM.tif]

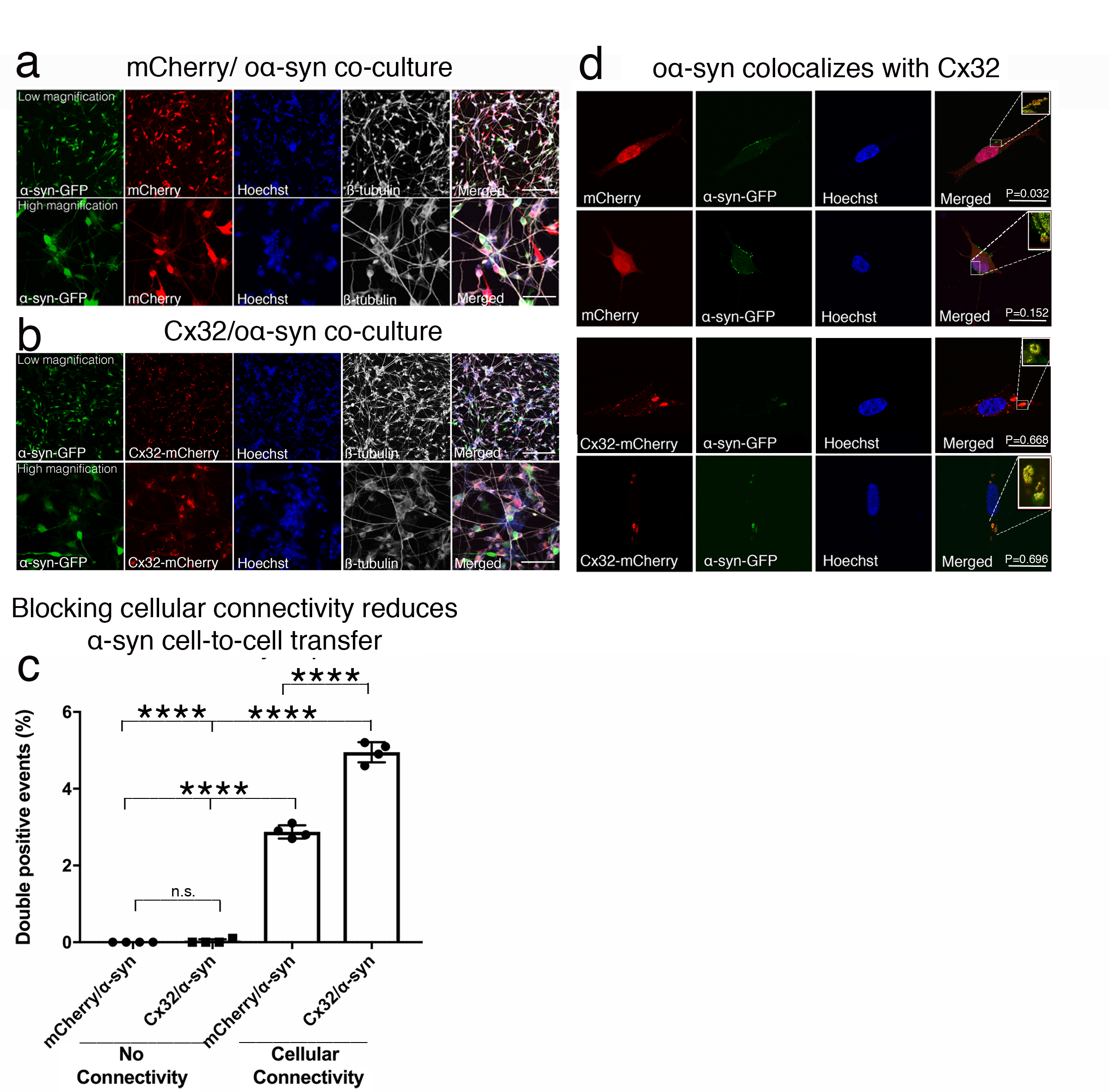

Supplement: Supplementary file 5 — Supplementary material 5 (TIFF 16683 kb) Suppl. Figure S2 (Online Resource 5). Cellular connectivity increases α-syn cell-to-cell transfer. a Representative confocal micrographs of differentiated SH-SY5Y donor and recipient co-cultures expressing α-syn-GFP (donor cells, green) and mCherry (acceptor cells, red), and immunolabeled with β3-tubulin antibodies (gray) and Hoechst (blue). b Differentiated SH-SY5Y co-cultures expressing α-syn-GFP (donor cells, green) and Cx32-mCherry (acceptor cells, red), and immunolabeled with β3-tubulin antibodies (gray) and Hoechst (blue). Scale bars represent 100 µm and 50 µm for low and high magnification, respectively. c Flow cytometry of undifferentiated SH-SY5Y cells, depicting α-syn-GFP transfer after 5 days (n = 4, independent experiments, one-way ANOVA, F(3, 12) = 910, n.s. = non-significant, ****p < 0.0001. d Confocal image analysis of FACS-sorted recipient cells expressing either mCherry or Cx32-mCherry, and containing α-syn-GFP oligomers, analyzed with HuygensPro to indicate a clear colocalization between Cx32 and α-syn-GFP, Pearson correlation coefficient of P = 0.032 and 0.159 for α-syn-GFP and mCherry compared to P = 0.668 and 0.696 for Cx32-mCherry and α-syn-GFP. [file 401_2019_2007_MOESM5_ESM.tif]

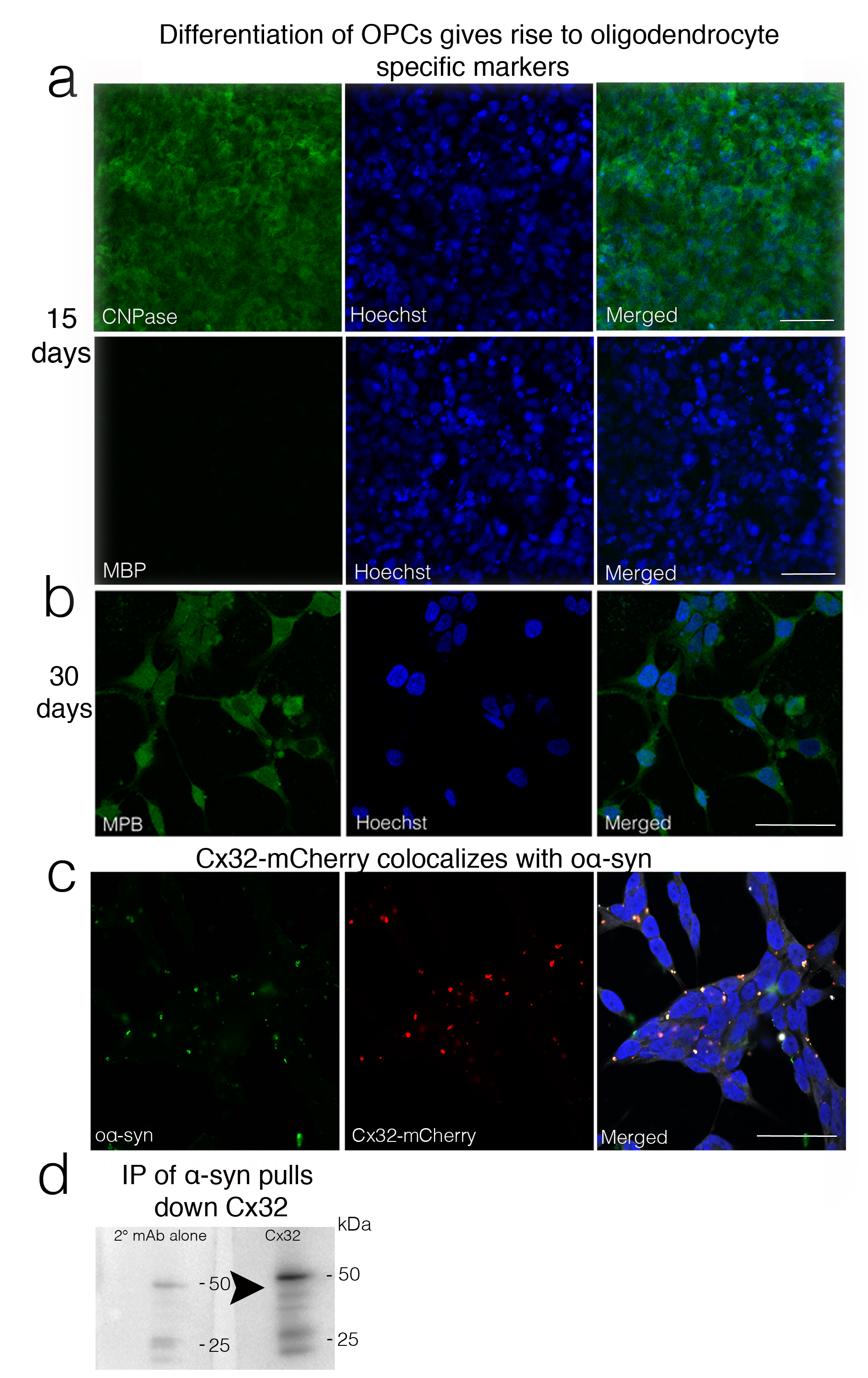

Supplement: Supplementary file 6 — Supplementary material 6 (TIFF 16647 kb) Suppl. Figure S3 (Online Resource 6). Cx32-mCherry colocalizes with oα-syn in human cells of the oligodendrocyte lineage.a Confocal image analysis of OPCs undergoing differentiation into mature oligodendrocytes, expressing the oligodendrocyte-specific marker CNPase (green) but lack MBP expression at 15 days of differentiation followed by Hoechst staining (blue). b Confocal image analysis of OPCs on day 30 of differentiation into mature oligodendrocytes, expressing the oligodendrocyte-specific marker MBP (green) and stained with Hoechst (blue). c Confocal image analysis of human OPCs (CNPase + , gray) overexpressing Cx32-mCherry (red) shows a high degree of colocalization as noted by the merging of Cx32-mCherry (red) and oα-syn-ATTO-488 (green); stained with Hoechst (blue). Scale bars represent 50 µm. d IP of oα-syn followed by Western blotting with Cx32 antibody shows an interaction between Cx32 (arrow) and oα-syn in human OPCs. [file 401_2019_2007_MOESM6_ESM.tif]

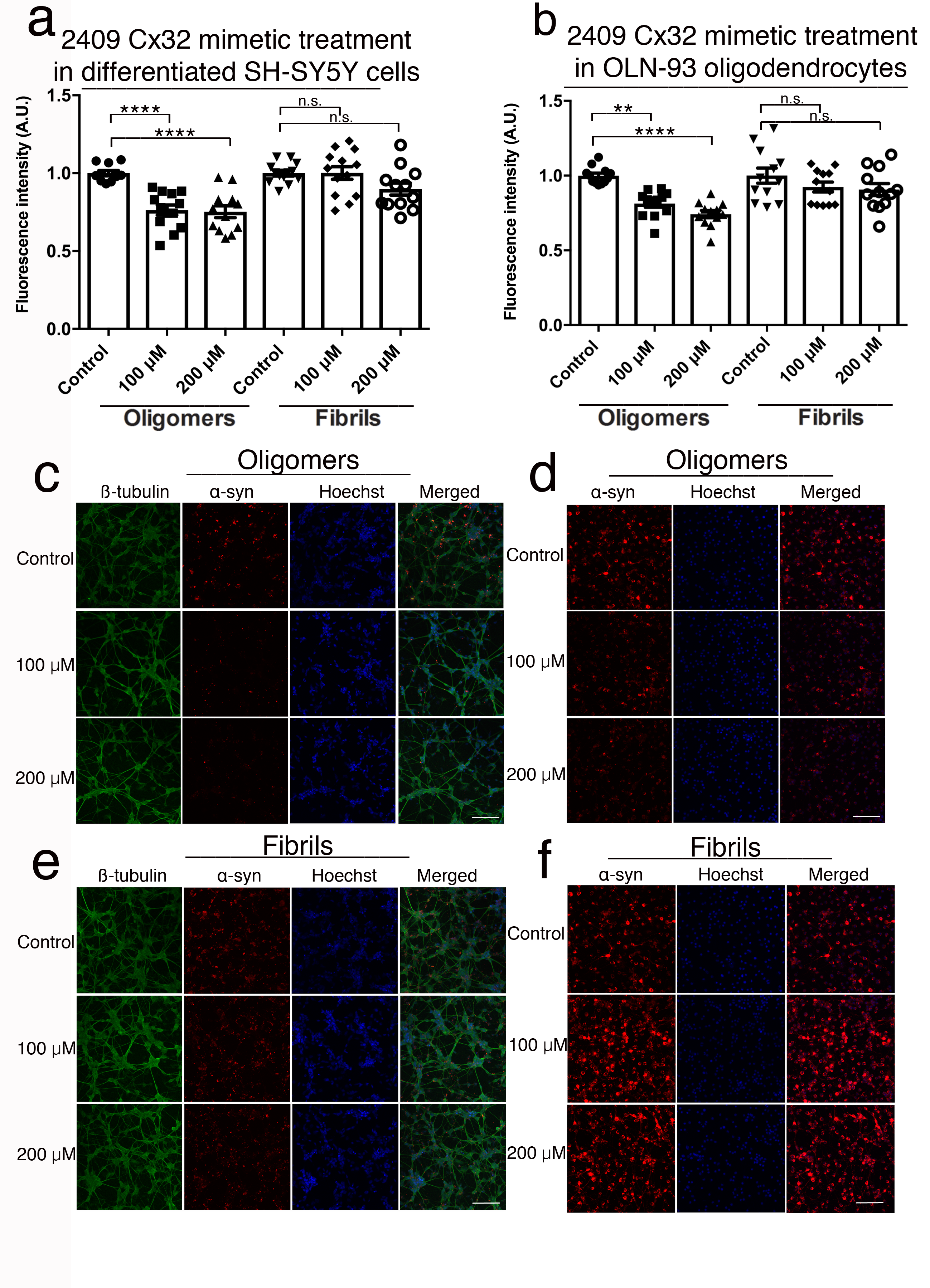

Supplement: Supplementary file 7 — Supplementary material 7 (TIFF 5126 kb) Suppl. Figure S4 (Online Resource 7). Cx32 peptide mimetics block oα-syn uptake but not fibrillar assemblies.a Fluorescence intensity measurements of α-syn-ATTO-550 uptake in differentiated SH-SY5Y cells exposed to oα-syn or fibrillar assemblies in the presence of Gap2409 peptide mimetics, (n = 12, one-way ANOVA followed by Tukey’s post hoc test for multiple comparisons, n.s; no significance, F(5, 68) = 13.33, ****p < 0.0001). b Fluorescence intensity measurements of α-syn-ATTO-550 uptake in differentiated OLN-93 oligodendrocyte cells exposed to oα-syn or fibrillar assemblies in the presence of Gap2409 peptide mimetics (n = 12, one-way ANOVA followed by Tukey’s post hoc test for multiple comparisons, n.s; no significance, F(5, 66) = 9.392, **p < 0.01, ****p < 0.0001). c Representative confocal micrographs of differentiated SH-SY5Y cells exposed to oligomeric assemblies. (d) Representative confocal micrographs of differentiated OLN-93 oligodendrocyte cells exposed to oligomeric assemblies. e Representative confocal micrographs of differentiated SH-SY5Y cells exposed to fibrillar assemblies f Representative confocal micrographs of differentiated OLN-93 oligodendrocyte cells exposed to fibrillar assemblies. Cells were immunolabeled with the neuron-specific β3-tubulin (green), α-syn-ATTO-550 (red), Hoechst (blue) and visualized using confocal microscopy. Scale bars represent 100 µm. [file 401_2019_2007_MOESM7_ESM.tif]

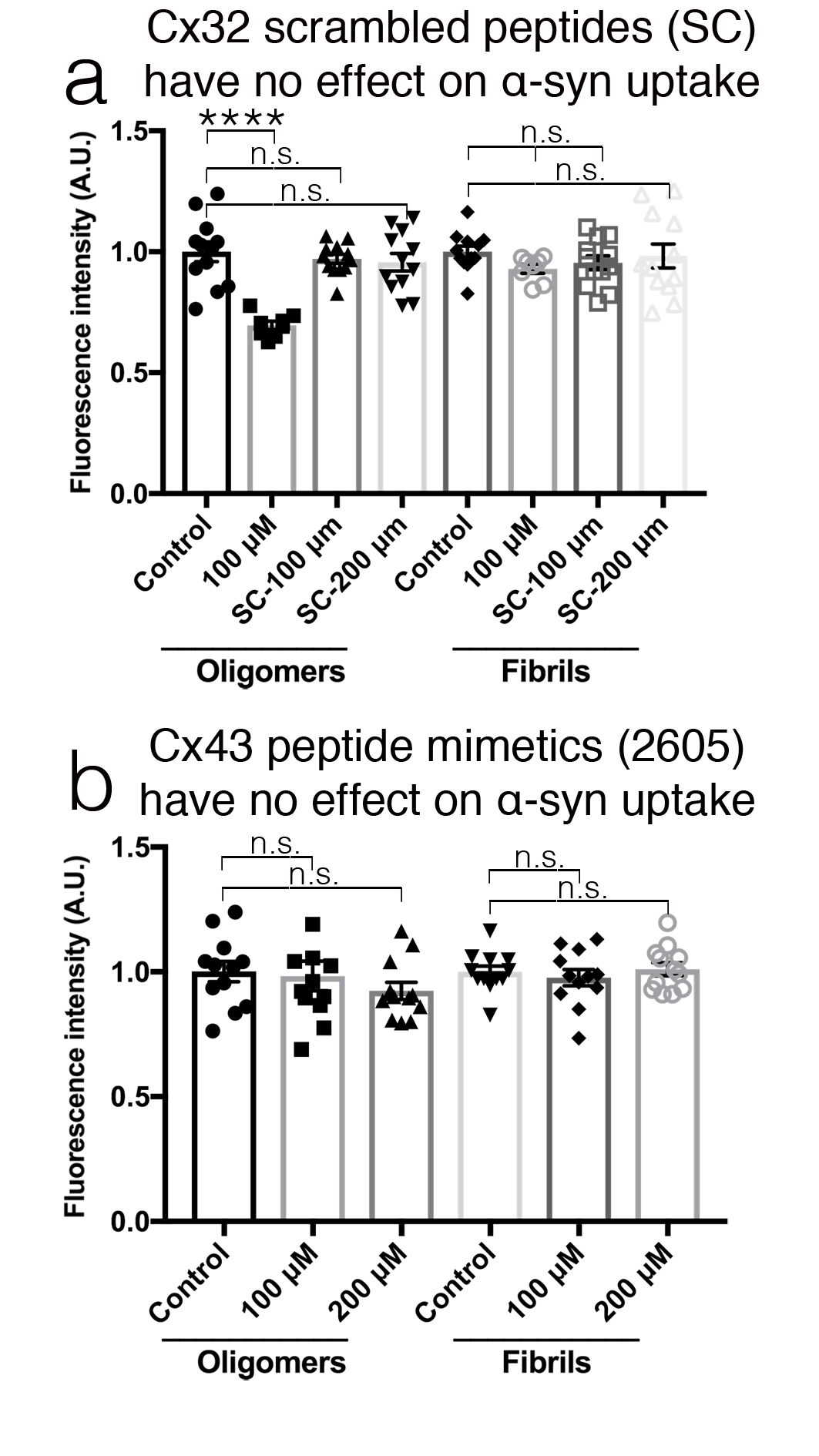

Supplement: Supplementary file 8 — Supplementary material 8 (TIFF 5920 kb) Suppl. Figure S5 (Online Resource 8). Scrambled Cx32 or Cx43 peptide mimetics have no effect on oligomeric or fibrillar α-syn uptakea Fluorescence intensity measurements of α-syn-ATTO-550 uptake in differentiated human SH-SY5Y cells exposed to oα-syn or fibrillar assemblies in the presence of functional and scrambled (SC) Gap3211 peptide mimetics, (n = 12, one-way ANOVA followed by Tukey’s post hoc test for multiple comparisons, n.s; no significance, F(7, 80) = 7.044, ****p < 0.0001). b Fluorescence intensity measurements of α-syn uptake in differentiated SH-SY5Y cells exposed to oα-syn or fibrillar assemblies in the presence of functional Cx43 (Gap2605) peptide mimetics, (n = 12, one-way ANOVA followed by Tukey’s post hoc test for multiple comparisons, n.s; no significance; F(5, 66) = 0.6833). [file 401_2019_2007_MOESM8_ESM.tif]

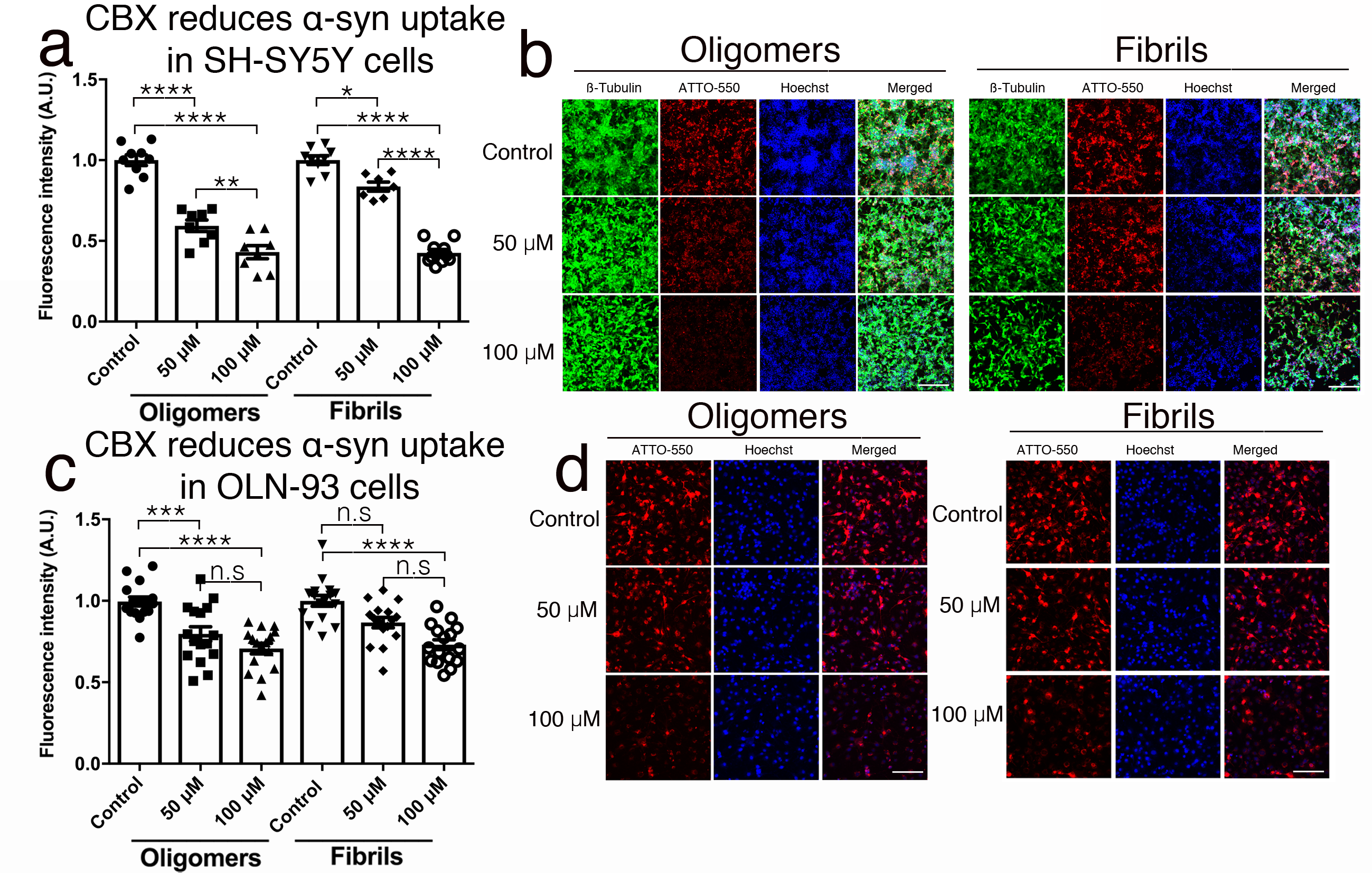

Supplement: Supplementary file 9 — Supplementary material 9 (TIFF 3158 kb) Suppl. Figure S6 (Online Resource 9). The pan-Cx specific gap junction inhibitor carbenoxolone (CBX) blocks the uptake of oligomeric and fibrillar α-syn assembliesa Fluorescence intensity measurements of α-syn-ATTO-550 uptake in differentiated human SH-SY5Y cells exposed to oα-syn or fibrillar assemblies in the presence of increasing concentrations of the pan-specific gap junction inhibitor CBX (n = 9, of 3 independent experiments one-way ANOVA followed by Tukey’s post hoc test for multiple comparisons, n.s; no significance, F(5, 45) = 76.6, *p < 0.05, **p < 0.01, ****p < 0.0001). b Representative confocal micrographs of differentiated SH-SY5Y cells co-labeled with β3-tubulin (green), α-syn (ATTO-550) and Hoechst (blue) visualized using confocal microscopy. c Fluorescence intensity measurements of oα-syn and fibrillar uptake in differentiated OLN-93 oligodendrocytes in the presence of CBX (n = 16 in 4 independent experiments, one-way ANOVA followed by Tukey’s post hoc test for multiple comparisons, n.s; no significance, F(5, 90) = 14.31, ***p < 0.001, ****p < 0.0001). d Representative confocal micrographs of differentiated OLN-93 oligodendrocytes co-labeled with α-syn (ATTO-550) and Hoechst (blue) visualized using confocal microscopy. Scale bars represent 100 µm. [file 401_2019_2007_MOESM9_ESM.tif]

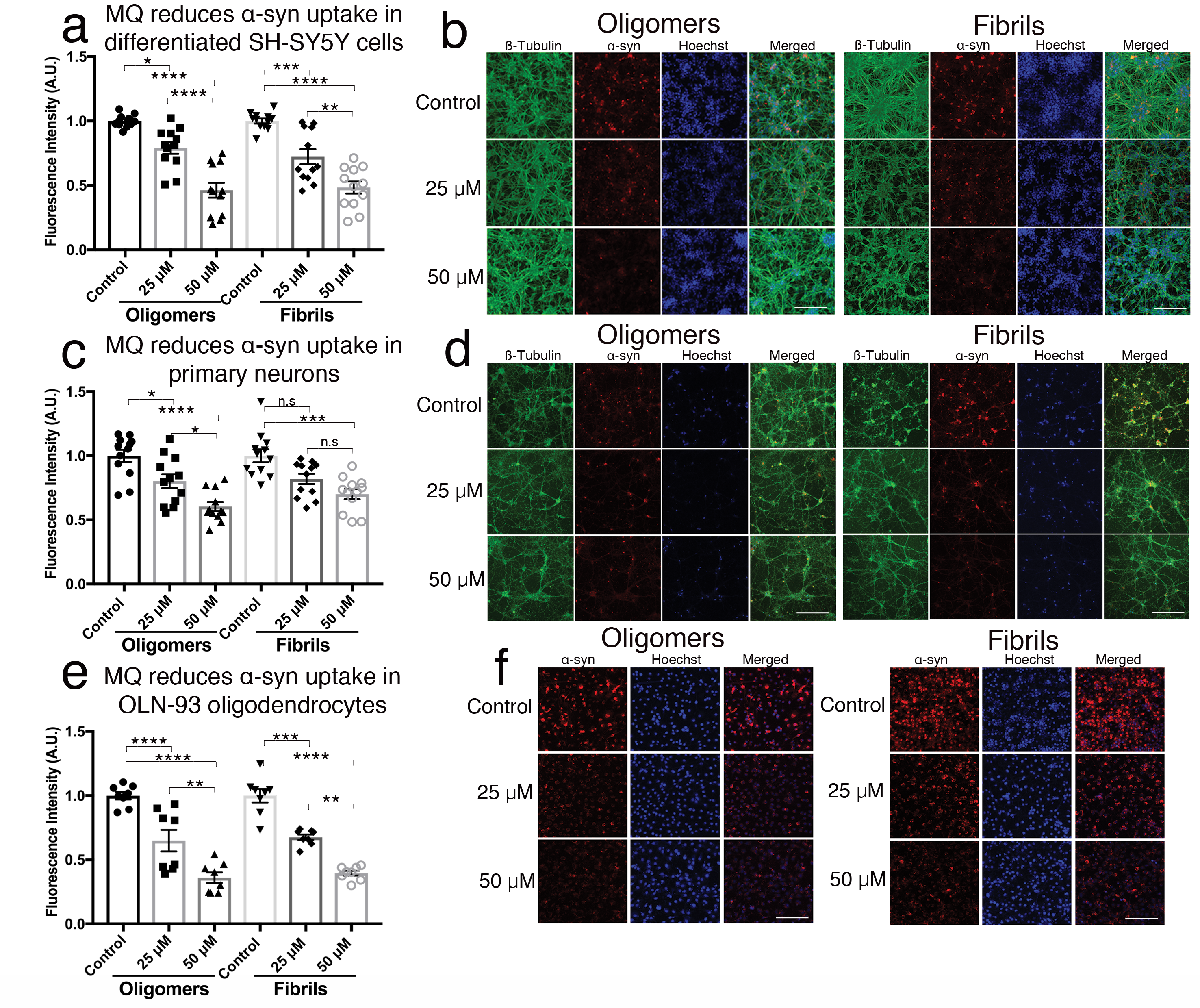

Supplement: Supplementary file 10 — Supplementary material 10 (TIFF 7483 kb) Suppl. Figure S7 (Online Resource 10). The selective gap junction inhibitor mefloquine (MQ) blocks the uptake of oligomeric and fibrillar α-syn assemblies.a Fluorescence intensity measurements of oα-syn and fibrillar uptake in differentiated SH-SY5Y cells in the presence of the selective gap junction inhibitor MQ, (n = 12, one-way ANOVA followed by Tukey’s post hoc test for multiple comparisons, F(5, 66) = 29.18, *p < 0.05, **p < 0.01, ***p < 0.001, ****p < 0.0001). b Representative micrographs of differentiated human SH-SY5Y cells co-labeled with β3-tubulin (green), α-syn-ATTO-550 (red) and Hoechst (blue) visualized under confocal microscopy. c Fluorescence intensity measurements of oα-syn and fibrillar uptake in primary cortical neurons (DIV 10) in the presence of MQ, (n = 12, one-way ANOVA followed by Tukey’s post hoc test for multiple comparisons, n.s; no significance, F(5, 66) = 12.46, *p < 0.05, ***p < 0.001, ****p < 0.0001). d Representative micrographs of primary cortical neurons co-labeled with β3-tubulin (green), α-syn-ATTO-550 (red) and Hoechst (blue). e Fluorescence intensity measurements of oα-syn and fibrillar uptake in differentiated OLN-93 oligodendrocytes in the presence of MQ, (n = 12, one-way ANOVA followed by Tukey’s post hoc test for multiple comparisons, F(5, 42) = 35.33, **p < 0.01, ***p < 0.001, ****p < 0.0001). Representative confocal micrographs of differentiated OLN-93 oligodendrocytes co-labeled with α-syn-ATTO-550 (red) and Hoechst (blue) visualized using confocal microscopy. Scale bars represent 100 µm. [file 401_2019_2007_MOESM10_ESM.tif]

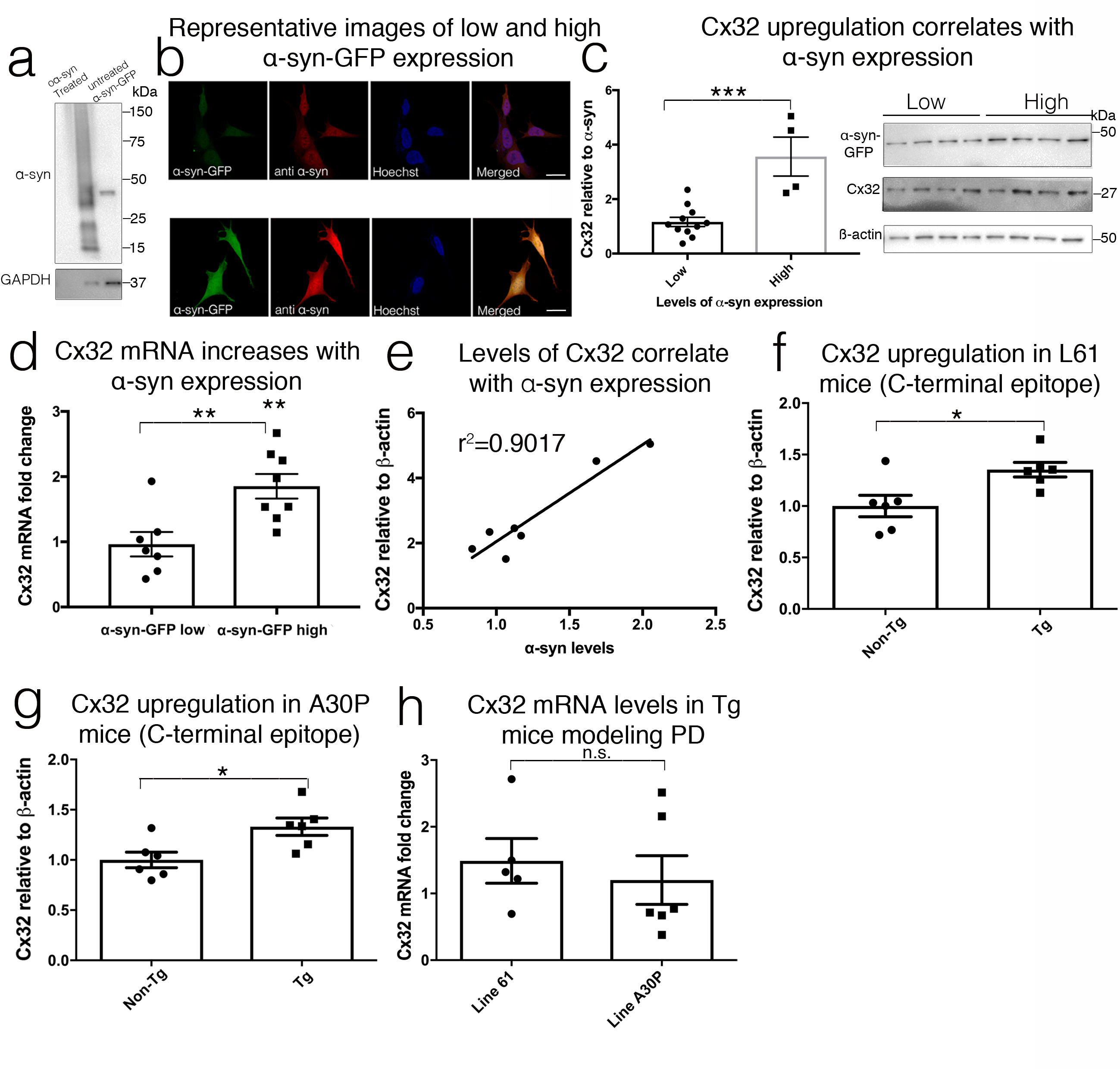

Supplement: Supplementary file 11 — Supplementary material 11 (TIFF 938 kb) Suppl. Figure S8 (Online Resource 11). Cx32 upregulation correlates with human α-syn expression.a Western blot of differentiated SH-SY5Y cells expressing α-syn-GFP exposed to oα-syn assemblies shows the uptake of oα-syn assemblies and the lack of α-syn-GFP degradation in untreated α-syn-GFP cells. b Confocal micrographs of low- and high-expression α-syn-GFP cells sorted by FACS, and co-labeled with α-syn antibody (red) and Hoechst (blue). Scale bars represent 20 µm. c Western blot analysis of Cx32 protein levels in differentiated SH-SY5Y cells expressing low (n = 11) or high α-syn-GFP (n = 4, unpaired, two-tailed t test, t13 = 4.862, ***p < 0.001). d Cx32 mRNA fold change of differentiated SH-SY5Y cells expressing low (n = 7) and high levels (n = 8) of α-syn-GFP and wild-type non-transfected cells as controls (one-way ANOVA followed by Tukey’s post hoc test for multiple comparisons, F(2, 16) = 11.81 **p < 0.007). e Correlation analysis between the levels of α-syn and Cx32 protein expression in differentiated SH-SY5Y cells (R2 = 0.9017, ***p < 0.001). f Western blot analysis of Cx32 proteins in non-Tg and Tg cohorts expressing human wild-type α-syn (L61, n = 5, Mann–Whitney test U = 5, n1 = n2 = 5 *p < 0.05). g Western blot analysis of Cx32 in non-Tg and Tg cohorts expressing human α-syn proteins harboring the A30P mutation (A30P) using C-terminal region antibodies to Cx32 (Mann–Whitney test U = 3, n1 = n2 = 6 *p < 0.05). h Cx32 mRNA fold change of non-Tg and Tg-L61 α-syn (n = 5) or mutant-A30P α-syn and age-matched control cohorts (n = 6, one-way ANOVA followed by Tukey’s post hoc test for multiple comparisons, n.s; no significance, F(3, 18) = 0.4887). [file 401_2019_2007_MOESM11_ESM.tif]

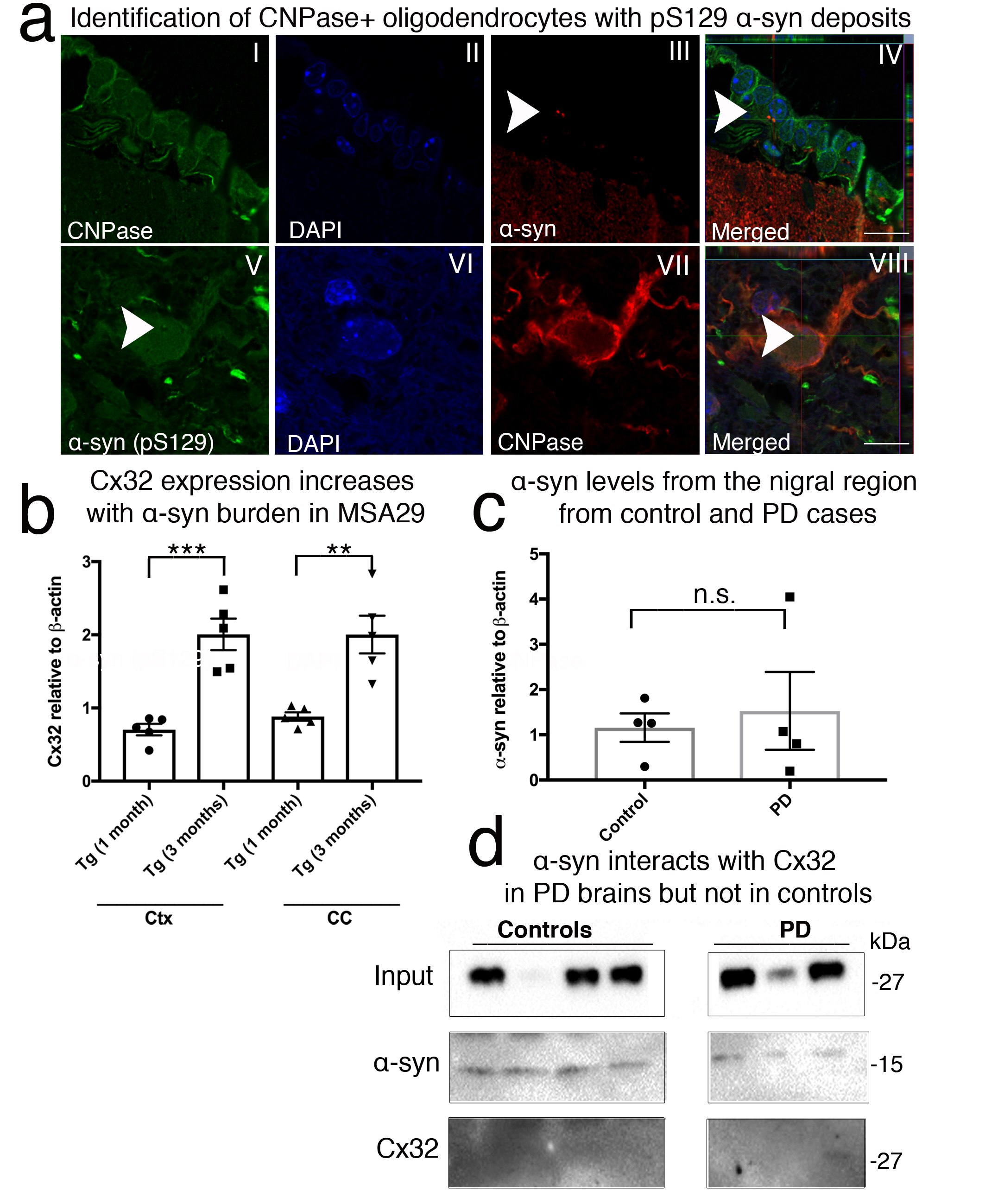

Supplement: Supplementary file 12 — Supplementary material 12 (TIFF 16224 kb) Suppl. Figure S9 (Online Resource 12). Cx32 interacts with α-syn in human PD brains.a Confocal image analysis of tissue sections from aged A30P mice immunolabeled with the oligodendrocyte-specific antibody CNPase (green), and α-syn (red; I–IV), or CNPase (red) and α-syn phosphorylated at serine-129 (green; V–VIII), scale bar 10 µm. b. Densitometric analysis of Cx32 protein levels in young ~ 1-month (3 weeks) and adult Tg (3 months) MBP29 cohorts from the cortex and corpus callosum (n = 5, one-way ANOVA followed by Tukey’s post hoc test for multiple comparisons, F(3, 16) = 16.13, **p < 0.01, ***p < 0.001). c Densitometry analysis of human α-syn within the nigra of control and PD cases (unpaired two-tailed t test, n.s. = no significance). d IP of α-syn from human PD cases and age-matched controls followed by Western blot analysis. Note that Cx32 is identified in PD cases (total: 2 out of 4) but not in controls (total: 0 out 4). [file 401_2019_2007_MOESM12_ESM.tif]
